# A fully human IgG1 anti-PD-L1 MAb in an *in vitro* assay enhances antigen-specific T-cell responses

**DOI:** 10.1038/cti.2016.27

**Published:** 2016-05-20

**Authors:** Italia Grenga, Renee N Donahue, Lauren M Lepone, Jacob Richards, Jeffrey Schlom

**Affiliations:** 1 Laboratory of Tumor Immunology and Biology, Center for Cancer Research, National Cancer Institute, National Institutes of Health, Bethesda, MD, USA

## Abstract

Monoclonal antibodies (MAbs) that interfere with checkpoint molecules are being investigated for the treatment of infectious diseases and cancer, with the aim of enhancing the function of an impaired immune system. Avelumab (MSB0010718C) is a fully human IgG1 MAb targeting programmed death-ligand 1 (PD-L1), which differs from other checkpoint-blocking antibodies in its ability to mediate antibody-dependent cell-mediated cytotoxicity. These studies were conducted to define whether avelumab could enhance the detection of antigen-specific immune response in *in vitro* assays. Peripheral blood mononuclear cells from 17 healthy donors were stimulated *in vitro*, with and without avelumab, with peptide pools encoding for cytomegalovirus, Epstein–Barr virus, influenza and tetanus toxin or the negative peptide control encoding for human leukocyte antigen. These studies show for the first time that the addition of avelumab to an antigen-specific IVS assay (a) increased the frequency of activated antigen-specific CD8^+^ T lymphocytes, and did so to a greater extent than that seen with commercially available PD-L1-blocking antibodies, (b) reduced CD4^+^ T-cell proliferation and (c) induced a switch in the production of Th2 to Th1 cytokines. Moreover, there was an inverse correlation between the enhancement of CD8^+^ T-cell activation and reduction in CD4^+^ T-cell proliferation induced by avelumab. These findings provide the rationale for the use of avelumab anti-PD-L1 in *in vitro* assays to monitor patient immune responses to immunotherapies.

Monoclonal antibodies (MAbs) designed to interfere with checkpoint molecules, such as programmed death-1 (PD-1) and programmed death-ligand 1 (PD-L1), are currently being investigated for the treatment of infectious diseases and cancer.^1, 2, 3^ The immune system of patients with chronic viral infections or cancer is often impaired in function and is unable to mount an effective response against the virus or to recognize and eliminate malignant cells. The rationale of blocking the PD-1/PD-L1 pathway in these settings is to reduce or eliminate immunosuppressive signals between immune cells and infected or tumor cells.^4^ The addition of checkpoint inhibitors to *in vitro* and *in vivo* models of infectious disease enhances immune activation and reduces viral load.^5, 6, 7^ On the basis of these results, clinical studies are ongoing using an anti-PD-L1 antibody (NCT02028403) and anti-PD-1 antibody (NCT02408861) in patients with HIV, and an anti-PD-1 antibody in patients with hepatitis C virus (NCT01658878, NCT00703469). Blockade of the PD-1/PD-L1 pathway has also become a major focus in anticancer drug development, with the US Food and Drug Administration granting approval of several antibodies blocking immune checkpoints for the treatment of advanced melanoma, Hodgkin's lymphoma, and lung and bladder cancer.^8, 9, 10^ Blockade of the PD-1/PD-L1 pathway is also being actively examined in a number of cancers that are often associated with chronic viral infections, such as hepatocellular carcinoma (NCT01658878), cervical cancer (NCT02291055, NCT02164461) and anal cancer (NCT01671488).

Most of the antibodies targeting PD-1 or PD-L1 in clinical development are fully human or humanized, and are either of the IgG4 isotype, which does not mediate antibody-dependent cell-mediated cytotoxicity (ADCC), or of the IgG1 isotype and are specifically engineered to eliminate ADCC activity, to avoid potential toxicity against immune cells that may express the target antigen. ADCC, however, is implicated in the mechanism of action of several widely used MAbs,^11^ including trastuzumab, which targets Her2/neu on metastatic breast cancer cells,^12^ rituximab, which targets CD20 on lymphoma cells,^13^ and cetuximab, which targets epidermal growth factor receptor on KRAS wild-type colorectal and squamous cell cancer of the head and neck cells.^14^ Although each of the molecules targeted by these agents is expressed on non-target cell populations, all three of these MAbs have demonstrated safety and clinical benefit, and are approved by the US Food and Drug Administration for their respective indications.

MSB0010718C (avelumab) is a fully human IgG1 antibody targeting PD-L1 that is capable of mediating ADCC of tumor cells.^15^ A phase I dose escalation and expansion study of avelumab in 117 patients with advanced cancer has recently been completed at the NIH Clinical Center (NCT01772004). Preliminary results showed clinical efficacy in terms of prolonged disease stabilization and RECIST responses (manuscript in preparation), and a toxicity profile similar to that of other antibodies targeting PD-1 or PD-L1.^16, 17^ Evaluation of 123 immune cell subsets in the peripheral blood mononuclear cells (PBMCs) of these patients 15, 43 and 127 days after initiation of avelumab treatment showed little, if any, change from pretreatment levels, including those subsets that expressed PD-L1 (manuscript in preparation).^16, 17, 18^ In addition, a recent *in vitro* study has shown that, whereas avelumab efficiently mediates ADCC of human tumor cells that express PD-L1, only minor levels of avelumab-mediated lysis were noted when unstimulated PBMCs were used as targets.^15^ Despite these studies that demonstrate no loss of PD-L1-expressing immune cells in patients treated with avelumab, and a lack of avelumab-mediated lysis of PBMCs in *in vitro* studies, there is concern by some that when immune cells are activated, and PD-L1 expression increases, avelumab may induce lysis of activated immune cells.

Recent studies have shown that blockade of the PD-1/PD-L1 pathway, using commercially available blocking antibodies, in PBMCs of patients with chronic infections of hepatitis C virus or HIV, can restore functionally impaired T-cell immune responses.^6, 19, 20, 21^ The current study examined the ability of blockade of the PD-1/PD-L1 pathway to enhance immune activation in a normally functioning immune system, using PBMCs from apparently healthy individuals, as well as investigated the potential lytic effects of avelumab on activated immune cells. We show for the first time that the addition of avelumab to an antigen-specific *in vitro* stimulation (IVS) assay using normally functioning peripheral immune cells (a) increased the frequency of activated antigen-specific CD8^+^ T lymphocytes, and did so to a greater extent than that seen with commercially available PD-L1-blocking antibodies, (b) induced a switch in the production of Th2 to Th1 cytokines, (c) reduced total cell number as well as CD4^+^ T lymphocyte frequency, which was attributed to a reduction in cell proliferation and (d) did not alter cell viability. Moreover, we show here that there was an inverse correlation between the enhancement of CD8^+^ T-cell activation and reduction in CD4^+^ T-cell proliferation induced by avelumab. In addition, the ability of avelumab to enhance antigen-specific immune activation in PBMCs from metastatic breast cancer patients was demonstrated. Overall, these findings indicate that avelumab did not induce lysis of activated human immune cells that express elevated levels of PD-L1, but instead significantly increased antigen-specific immune activation, and support the use of avelumab anti-PD-L1 in *in vitro* assays to monitor patient immune responses to immunotherapies.

## Results

### Analysis of antigen-specific T-cell responses employing different anti-PD-L1 MAbs

**Figure 1:**
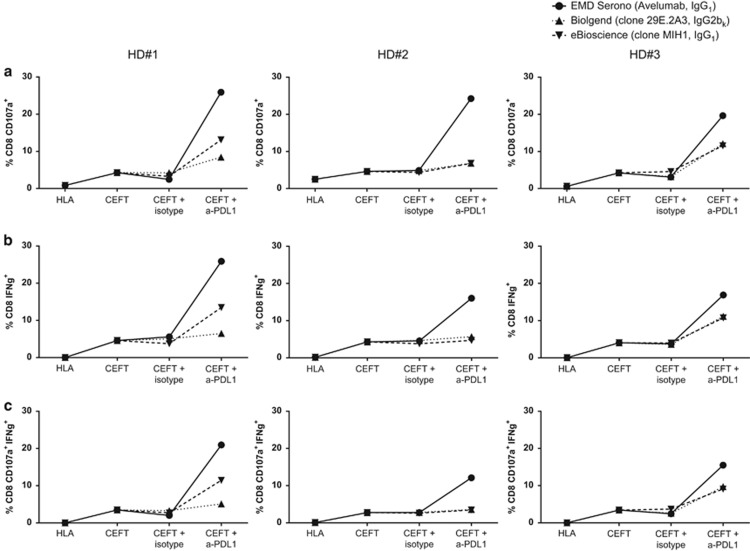
Anti-PD-L1 (avelumab) enhances IFNγ production and CD107a positivity in CD8^+^ T lymphocytes to a greater extent than other commercially available anti-PD-L1-blocking antibodies. PBMCs from three HD were stimulated in an IVS assay as described in Supplementary Figure 3a for 7 days with peptide pools of CEFT (containing peptides encoding for CMV, EBV, Flu and tetanus toxin, 0.1 μg ml^−1^) or the negative control HLA, and were treated with 20  μg ml^−1^ of EMD Serono anti-PD-L1 (avelumab, IgG1), eBioscience anti-PD-L1 (clone MIH1, IgG1), Biolegend anti-PD-L1 (clone 29E.2A3, IgG2b_k_) or the appropriate isotype controls; untreated cultures also served as controls. Cells were then rested for 4 days and restimulated overnight with the same peptides in the presence of anti-CD107a, brefeldin A and monensin, and were assessed by intracellular cytokine staining. Frequency of PBMCs on day 12 that were CD8^+^CD107a^+^ (**a**), CD8^+^IFNγ^+^ (**b**) and CD8^+^CD107a^+^IFNγ^+^ (**c**). Each line represents a different anti-PD-L1 antibody.

We first investigated whether the addition of different anti-PD-L1-blocking MAbs would differentially enhance the magnitude of antigen-specific responses in *in vitro* assays. PBMCs from three healthy donors (HDs) were stimulated with peptide pools encoding for cytomegalovirus (CMV), Epstein–Barr virus (EBV), influenza (Flu) and tetanus toxin (designated hereinafter as CEFT) or with a negative control peptide pool encoding for human leukocyte antigen (HLA). Cells were then treated with anti-PD-L1 avelumab (IgG1, EMD Serono, Billerica, MA, USA), anti-PD-L1 clone MIH1 (IgG1, eBioscience, San Diego, CA, USA), anti-PD-L1 clone 29E.2A3 (IgG2b,k, Biolegend, San Diego, CA, USA) or the corresponding isotype controls. All three donors analyzed displayed a modest response to CEFT peptides relative to the negative control HLA, as demonstrated by an increase in CD8^+^CD107a^+^ T cells (Figure 1a), CD8^+^ IFNγ^+^ T cells (Figure 1b) and CD8^+^CD107a^+^ IFNγ^+^ T cells (Figure 1c). The addition of the anti-PD-L1-blocking antibodies increased the frequency of activated CD8^+^ T lymphocytes, with a wide range of responses noted among the three antibodies. For example, activated CD8^+^ T lymphocytes (those positive for CD107a, IFNγ or both CD107a and IFNγ) were increased up to sixfold with the addition of avelumab, 3.3-fold with the addition of the MIH1 and twofold with the addition of 29E.2A3 in HD #1, relative to cultures stimulated with CEFT and treated with the corresponding isotype control. In all three HDs, there was a markedly greater increase in activated CD8^+^ T lymphocytes in cultures treated with avelumab compared with those treated with the other two anti-PD-L1-blocking antibodies. No differences in CD8^+^ T-cell activation were noted when any anti-PD-L1-blocking antibody or the corresponding isotype control was added to PBMCs stimulated with the negative control peptide pool HLA.

**Figure 2:**
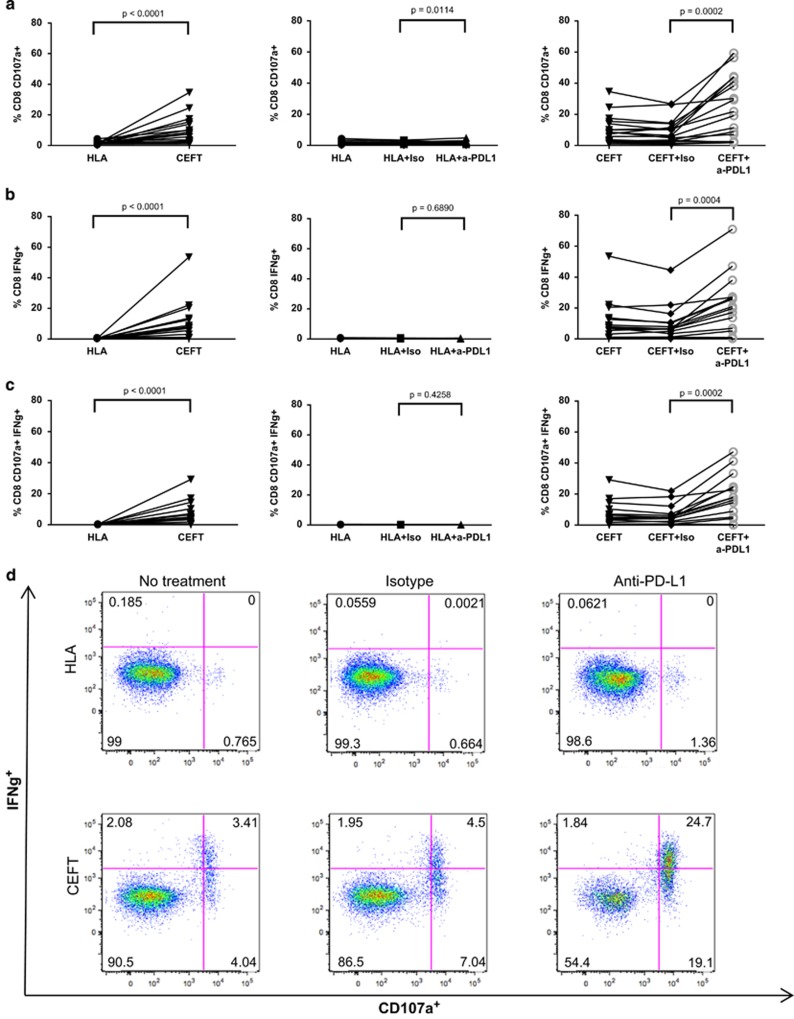
Anti-PD-L1 (avelumab) enhances the frequency of CD8^+^ T lymphocytes that produce IFNγ and are positive for the degranulation marker CD107a. PBMCs from 17 HDs were stimulated with CEFT or HLA and were treated with avelumab or the isotype control as described in Supplementary Figure 3a. Frequency of CD8^+^ T cells on day 12 that were CD107a^+^ (**a**), IFNγ^+^ (**b**) and CD107a^+^IFNγ^+^ (**c**). Each line represents an individual HD. Data were analyzed with the Wilcoxon matched-pairs signed rank test, with *P*-values indicated. (**d**) Representative dot plots of one HD on day 12. Values indicate the percentage of CD8^+^ T cells that produced IFNγ or were positive for CD107a.

On the basis of these results, we next examined the effects of avelumab in a greater number of HDs (*n*=17). As shown in Figure 2 (left column), there was a significant increase in CD8^+^ T cells expressing the degranulation marker CD107a, and CD8^+^ T cells producing IFNγ, as well as multifunctional CD8^+^ T cells (CD107a^+^ IFNγ^+^ ; *P<*0.0001), when donors were stimulated with the CEFT peptide pool compared with the negative control peptide pool, HLA. The addition of avelumab to CEFT-stimulated cultures significantly enhanced CD8^+^ T-cell activation compared with the isotype control, with increases noted in CD8^+^ T cells that were CD107a^+^ (*P*=0.0002, Figure 2a, right column), IFNγ^+^ (*P=*0.0004, Figure 2b, right column) and CD107a^+^ IFNγ^+^ (*P=*0.0002, Figure 2c, right column). In 12 out of 17 donors, the frequency of CD8^+^ CD107a^+^ IFNγ^+^ cells was between two- to ninefold greater than that seen with the isotype control when cultures were stimulated with CEFT and treated with avelumab. In addition, no marked changes in the frequency of activated CD8^+^ T cells were noted when avelumab was added to cultures stimulated with the negative control peptide pool HLA (Figures 2a–c, middle column), demonstrating that, although avelumab enhances antigen-specific immune responses, it does not enhance nonspecific immune activation. No differences were observed between cultures that were stimulated with CEFT or HLA and treated with the isotype control, compared with cultures that were stimulated with CEFT or HLA and not treated with any antibody. Representative dot plots from one HD are shown, demonstrating the frequency of CD8^+^ T lymphocytes that were positive for IFNγ, CD107a or IFNγ and CD107a (Figure 2d). In this example, the addition of avelumab increased the frequency of antigen-specific multifunctional CD8^+^ T cells (CD107a^+^IFNγ^+^) by more than fivefold, relative to the isotype control in CEFT-stimulated PBMCs. Activation of CD4^+^ T lymphocytes, measured by cytokine production or CD107a positivity, was not enhanced when cultures were stimulated with CEFT and treated with avelumab.

To examine whether avelumab similarly enhances the development of antigen-specific responses in cancer patients, PBMCs from patients with metastatic breast cancer (*n*=7) were stimulated with CEFT or HLA peptide pools in the presence or absence of avelumab, and were assessed for the development of antigen-specific responses in CD8^+^ T cells. As with the HDs, avelumab enhanced antigen-specific responses in a subset of the cancer patients, with three out of seven patients displaying an increase in CD8^+^ T cells that produced IFNγ and/or expressed the degranulation marker CD107a. Representative dot plots from one cancer patient are shown (Supplementary Figure 1), indicating multifunctional CD8^+^ T cells and their increase following avelumab treatment compared with the isotype control.

**Figure 3:**
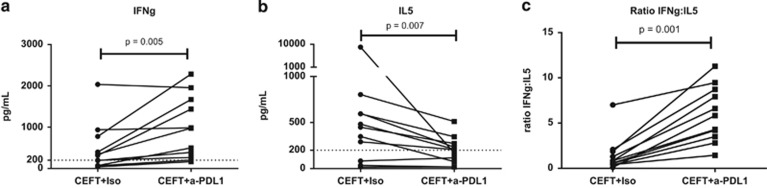
Anti-PD-L1 (avelumab) induces a switch in the production of Th2 to Th1 cytokines in PBMCs stimulated with CEFT. PBMCs from 11 HDs were stimulated with CEFT or HLA and were treated with avelumab or the isotype control as described in Supplementary Figure 3a. Supernatants collected on day 7 of the IVS assay were analyzed using the cytokine bead array for the production of Th1 (IFNγ, TNF, IL2) and Th2 (IL5, IL10, IL4) cytokines. Data represent the production of IFNγ (**a**), IL5 (**b**) and the ratio of IFNγ:IL5 (**c**) in PBMCs stimulated with CEFT and treated with avelumab or the isotype control. The dashed line indicates the level of detection of the assay. Among the cytokines tested, only IFNγ and IL5 were detectable. Data were analyzed with the Wilcoxon matched-pairs signed rank test, with *P*-values indicated.

To examine whether avelumab induces a switch in the production of Th2 to Th1 cytokines, we analyzed the supernatants of PBMCs from 11 HDs who were stimulated with CEFT and were treated with avelumab or the appropriate isotype control, using a cytokine bead array. Compared with the isotype control, in CEFT-stimulated PBMCs treated with avelumab, there was a significant increase in the Th1 cytokine IFNγ (*P=*0.005, Figure 3a) and a significant reduction in the Th2 cytokine IL5 (*P=*0.007, Figure 3b); this resulted in a statistically significant increase in the ratio of IFNγ:IL5 (*P=*0.001, Figure 3c). The other cytokines measured in this assay (Th1: TNF and IL2, and Th2: IL10 and IL4) were below the limits of detection.

### Association between the expression of PD-L1 and PD-1 on immune cells and the response to avelumab (anti-PD-L1)

**Figure 4:**
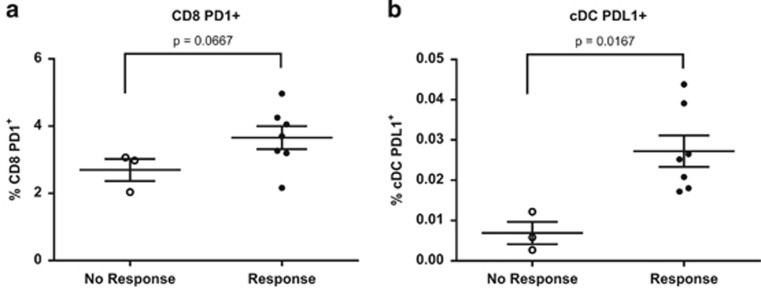
Expression of PD-1 and PD-L1 in immune subsets before stimulation has an impact on the immune response generated following stimulation with CEFT peptide pool and treatment with anti-PD-L1 (avelumab). Data represent the percentage of PBMCs that were (**a**) CD8^+^ T cells expressing PD-1 or (**b**) conventional dendritic cells (cDCs) expressing PD-L1 before stimulation with CEFT peptide pool and treatment with avelumab as described in Supplementary Figure 3a. Phenotype was assessed on day 0 after overnight rest of the IVS assay in seven HDs who demonstrated an enhanced immune response (R) and in three HDs without an enhanced immune response (NR), following stimulation with CEFT and treatment with avelumab. Data were analyzed with the Mann–Whitney test, with *P*-values indicated.

Although the addition of avelumab to an IVS assay enhanced CD8^+^ T-cell activation in PBMCs from the majority (>75%) of HDs, we noted that there were some donors who did not respond to avelumab. We next analyzed PBMCs before the stimulation assay from seven HDs who responded and three who did not respond to avelumab for the frequency of standard immune cell subsets (CD4^+^ and CD8^+^ T lymphocytes, B lymphocytes, natural killer (NK) cells, NK-T cells, plasmacytoid dendritic cells, conventional dendritic cells, T regulatory cells (Tregs) and myeloid derived suppressor cells), as well as PD-1 and PD-L1 expression within each of these subsets. PBMCs were rested overnight to allow for the recovery of surface molecules potentially affected by cryopreservation.^22, 23^ Although there were no significant differences in immune phenotype before the stimulation assay with respect to the standard immune subsets, we found that a donor's immune phenotype in terms of expression of PD-1 and PD-L1 within certain subsets had an impact on whether a donor responded to avelumab. Compared with non-responders, responders had a trend of an increase in CD8^+^ T cells positive for PD-1 (*P*=0.0667, Figure 4a), and conventional dendritic cells that expressed PD-L1 (*P*=0.0167, Figure 4b). No additional differences between the responders and non-responders were observed in PD-1 and PD-L1 expression in the other immune cell subsets analyzed.

### Effect of avelumab on cell number, viability and immune cell subset frequency following the IVS assay with avelumab (anti-PD-L1)

Avelumab has been described to mediate ADCC against tumor cell lines; however, when non-activated human PBMCs were used as targets in an ADCC assay, nominal levels of ADCC were detected.^15^ To investigate the potential cytotoxic effects of avelumab on activated immune cells, PBMCs (2.5 × 10^6^) from HDs (*n*=17) were stimulated in an *in vitro* assay with CEFT or HLA peptides, and were treated with avelumab or the isotype control, and assessed for cell number and viability (Table 1). Cell number was increased a median of twofold (from 2.5 × 10^6^ to 5.05 × 10^6^ cells per well) in cultures stimulated with CEFT and treated with the isotype control, compared with a median increase of 1.8-fold (from 2.5 × 10^6^ to 4.6 × 10^6^ cells per well) when PBMCs were stimulated with CEFT and treated with avelumab; thus, cell number was slightly increased to a lesser extent when cultures were treated with avelumab compared with the isotype control (Table 1). In addition, relative to the isotype control, avelumab had no significant effect on cell viability when cultures were stimulated with either HLA (*P=*0.3529) or CEFT (*P=*0.2435) peptides (Table 1). We also investigated the effect of avelumab on the frequency of the following immune cell subsets at the end of the stimulation assay: CD4^+^ and CD8^+^ T lymphocytes, B lymphocytes, NK cells, NK-T cells, plasmacytoid dendritic cells, conventional dendritic cells, Tregs and myeloid derived suppressor cells. Among these subsets, there were significantly fewer CD4^+^ T lymphocytes when PBMCs were stimulated with CEFT or HLA peptide and treated with avelumab, compared with those treated with the isotype control; this occurred without any significant changes in cell viability. In contrast, avelumab had no marked effect on the frequency of total CD8^+^ T cells in cultures stimulated with CEFT (*P*=0.15); there was a trend of an increase in CD8^+^ T cells when cultures were stimulated with HLA (*P*=0.02). There was also a slight trend of an increase noted in B cells, when PBMCs were stimulated with CEFT peptides and treated with avelumab, compared with the isotype control (*P=*0.06); there were no alterations noted in the frequency of NK cells, NK-T cells or Tregs, and the percentages of conventional dendritic cells, plasmacytoid dendritic cells and myeloid derived suppressor cells were too low at the end of the stimulation assay to be evaluated.

**Figure 5:**
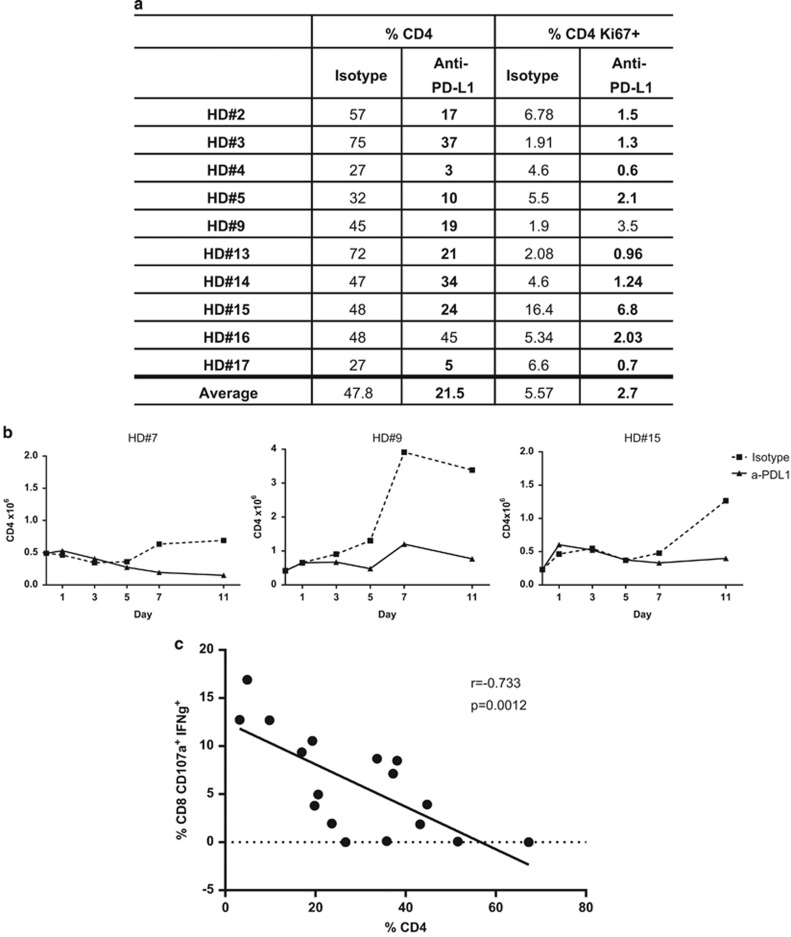
Anti-PD-L1 (avelumab) reduces CD4^+^ T lymphocyte proliferation. (**a**) PBMCs from HDs (*n*=9) stimulated with CEFT or HLA and treated with avelumab or the isotype control as described in Supplementary Figure 3a that had a decrease in CD4^+^ T-cell number were assessed on day 12 of the IVS assay with flow cytometry to determine the percentage of PBMCs that were CD4^+^ or CD4^+^Ki67^+^. Bold font highlights differences that are >25%, comparing PBMCs that were stimulated with CEFT peptides and treated with avelumab with PBMCs that were stimulated with CEFT and treated with the isotype control. (**b**) Absolute number of CD4^+^ T lymphocytes over time throughout the stimulation assay (calculated with flow cytometry and trypan blue exclusion) from PBMCs of three HDs that were stimulated with CEFT and treated with avelumab (solid line) or the isotype control (dashed line). (**c**) Correlation between the frequencies of CD4^+^ T lymphocytes and activated CD8^+^ T lymphocytes following anti-PD-L1 (avelumab) treatment in CEFT-stimulated PBMCs. PBMCs from 17 HDs stimulated with CEFT or HLA and treated with avelumab or the isotype control, as described in Supplementary Figure 3a, were assessed on day 12 of the IVS assay with flow cytometry to determine the percentage of PBMCs that were CD4^+^ and CD8^+^CD107a^+^IFNγ^+^. Values were calculated as a percentage of total PBMCs and were analyzed using the Spearman correlation.

To investigate the changes noted in CD4^+^ T lymphocytes, we assessed CD4^+^ T-cell proliferation at the end of the stimulation assay. We measured Ki67 expression in CD4^+^ T cells in PBMCs of nine HDs displaying a >25% reduction in CD4^+^ T-cell frequency; eight out of nine (89%) of these donors also displayed a reduction in CD4^+^Ki67^+^ cells when cultures were stimulated with CEFT peptides and treated with avelumab compared with the isotype control; this reduction in CD4^+^Ki67^+^ was statistically significant (*P=*0.0049; Figure 5a). CD4^+^ T-cell number was additionally examined throughout the stimulation assay (days 0, 1, 3, 5, 7 and 11) in three HDs stimulated with CEFT peptides and treated with avelumab or the isotype control to determine the temporal effects of avelumab on this population. As shown in Figure 5b, CD4^+^ T-cell number remained low in cultures stimulated with CEFT and treated with avelumab compared with those that were stimulated with CEFT peptides and treated with the isotype control, where cell number increased beginning on day 3 or 5 of the assay. These data demonstrate that cultures treated with avelumab displayed a reduction in the proliferation of CD4^+^ T lymphocytes.

To investigate the possibility that the changes in CD4^+^ T-cell frequency may be associated with ADCC, we measured PD-L1 and PD-1 in CD8^+^ and CD4^+^ T cells, and B cells, as well as in activated CD8^+^ T lymphocytes (producing IFNγ/positive for CD107a), at the end of the stimulation assay. Because of potential competition between avelumab and the antibody used to assess PD-L1 expression, PD-L1 was measured only in cultures stimulated with peptide pools (CEFT or HLA) in the absence of avelumab treatment. PD-1 and PD-L1 expression was increased in all subsets examined (CD4^+^ and CD8^+^ T cells, and B cells, as well as activated CD8^+^ T cells), with expression being the highest in B cells, when PBMCs were stimulated with CEFT peptide relative to those stimulated with the negative control peptide pool HLA (Table 2). These data suggest that CD4^+^ T lymphocytes would not likely be a target of avelumab-mediated ADCC relative to the other immune cell subsets based on their expression of PD-L1. Thus, all of the above results suggest that there is no cytotoxicity against CD4^+^ T cells, but instead a reduction in proliferation and expansion of this subset, when cultures were treated with avelumab in an IVS assay.

### Correlation between the reduced proliferation of CD4^+^ T lymphocytes and activation of CD8^+^ T lymphocytes following anti-PD-L1 treatment in CEFT peptide-stimulated PBMCs

On the basis of the notable increase in CD8^+^ T-cell activation and reduction in CD4^+^ T-cell proliferation noted above when PBMCs were stimulated with CEFT and treated with avelumab, we looked for possible correlations between CD8^+^ T-cell activation (CD107a^+^ IFNγ^+^) and the frequency of total CD4^+^ T cells. As shown in Figure 5c, there was an inverse correlation between multifunctional CD8^+^ T cells and total CD4^+^ T cells (*P=*0.0012, *r*=−0.733) when PBMCs were stimulated with CEFT and treated with avelumab. We also observed this phenomenon, but to a lesser extent, with other PD-L1-blocking antibodies, clone MIH1 and 29E.2A3 (Supplementary Figure 2).

### Effect of avelumab on gene expression in PBMCs stimulated with CEFT or HLA peptide pools

**Figure 6:**
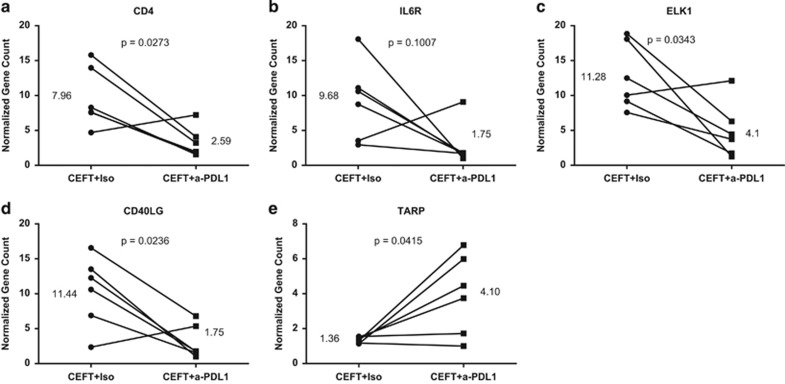
Notable immune-related genes identified with trends of differential expression between PBMCs of HDs that were stimulated with CEFT and treated with avelumab relative to the isotype control. PBMCs (*n*=6) at the end of the stimulation assay described in Supplementary Figure 3a (day 12) were lysed with RLT buffer; lysates containing 100 ng RNA were hybridized and run on the Nanostring nCounter Analysis System. Counts were background-subtracted and normalized according to the manufacturer's instructions. Fold changes were calculated using normalized medians of PBMCs that were stimulated with CEFT and treated with avelumab or the isotype control. Notable immune-related genes (**a**-**e**) were those identified with trends of differential expression (*P<*0.1 and sorted by fold change) between PBMCs of HDs stimulated with CEFT and treated with avelumab relative to the isotype control. Data were analyzed with the Wilcoxon matched-pairs signed rank test, with *P*-values and fold changes indicated.

To investigate whether there were any changes in gene expression that were associated with enhanced antigen-specific responses, PBMCs from HDs (*n*=6) and cancer patients (*n*=3) were stimulated with peptide pools (CEFT or HLA) and were exposed to avelumab or the isotype control and then examined by nanostring. A number of immune-related genes were significantly downregulated (Supplementary Table 1) or upregulated (Supplementary Table 2) in PBMCs of HDs following stimulation with CEFT peptides and treatment with avelumab compared with the isotype control. Notable changes included a threefold reduction in the CD4 transcript (Figure 6a), as well as reductions in molecules involved in Th2 signaling, including a 5.5-fold decrease in the interleukin 6 receptor (Figure 6b), and a 2.75-fold decrease in the E26 transformation-specific (ETS) domain-containing protein 1 (ELK1; Figure 6c), a transcription factor potentially involved in IL6 signaling. We also identified a significant reduction (6.5-fold) in the cluster of differentiation 40 ligand (CD40LG) in PBMCs of HDs stimulated with CEFT and treated with avelumab compared with the isotype control (Figure 6d). CD40LG is a member of the TNF superfamily that is expressed at various levels on antigen-presenting cells and shed from activated T lymphocytes and platelets; it has an immunosuppressive role in mediating immune suppression by myeloid derived suppressor cells and Tregs.^24, 25^ One notable increase in gene expression observed with avelumab compared with the isotype control (threefold increase) was seen with the TCR gamma alternate reading frame protein, which is found in some populations of mature T cells (Figure 6e). Several of the transcripts that were identified as being significantly altered in HDs were also similarly changed in PBMCs of cancer patients stimulated with peptide pools (CEFT) and treated with avelumab compared with the isotype control (data not shown).

## Discussion

The immune inhibitory role of the PD-1/PD-L1 axis is well defined in infectious diseases^19, 26, 27^ and cancer,^28, 29, 30^ with blockade of this pathway rescuing T-cell effector function. Several anti-PD-L1-blocking antibodies, including clones 29E.2A3 and MIH1, have demonstrated the ability to restore T-cell function (for example, proliferative/cytokine responses), when added to *in vitro* assays of PBMCs from patients with chronic infections such as HIV or hepatitis C virus that have defective T cells.^6, 19, 20, 21^ In the present study, we investigated the effect of blocking the PD-1/PD-L1 axis on T-cell activation using PBMCs from HDs, with apparently normal functioning immune systems. We compared the effect of avelumab, the first anti-PD-L1 antibody designed to mediate ADCC, to anti-PD-L1 clones 29E.2A3 and MIH1, which have been previously examined. We show for the first time that the addition of avelumab to an antigen-specific stimulation assay using viral peptide pools in PBMCs from HDs markedly increased the frequency of activated CD8^+^ T lymphocytes, identified by the production of IFNγ and the expression of the degranulation marker CD107a, and did so to a greater extent than the other PD-L1-blocking antibodies (Figures 1 and 2). We also demonstrate that avelumab enhanced the development of antigen-specific responses in a subset of patients (3/7) with metastatic breast cancer, albeit with a lower magnitude than that noted in HDs (Supplementary Figure 1). We also show for the first time that the addition of avelumab to an antigen-specific *in vitro* assay of HD PBMCs induced a switch in the production of Th2 to Th1 cytokines (Figure 3). These data were also supported at the gene level as indicated by a reduction of the receptor for interleukin 6 and the ETS domain-containing protein 1 (ELK1), which is a transcription factor potentially involved in IL6 signaling in CEFT-stimulated PBMCs treated with avelumab compared with the isotype control (Figures 6b and c). Blockade of the PD-1/PD-L1 pathway is reported to increase the production of Th1 cytokines,^22, 31^ and a recent study examining correlates of response to a different PD-L1 antibody (MPDL3280A) found that a tumor environment with a Th1 orientation (measured by gene expression) was associated with favorable clinical outcome.^32^ It has also been reported that the addition of a PD-1-blocking antibody to a stimulation assay of PBMCs from cancer patients decreased Th2 cytokines (for example, IL5 and IL13);^33^ however, those effects were not noted in HDs. Thus, our study is the first demonstrating a switch in the production of Th2 to Th1 cytokines with an anti-PD-L1-blocking antibody in a T-cell stimulation assay of PBMCs from HDs using a viral peptide pool consisting of mostly major histocompatibility complex class I peptides.

As avelumab is a fully human IgG1 anti-PD-L1 antibody that is capable of mediating ADCC, concern has been raised by some that this agent may induce depletion of immune cells that express PD-L1; however, a number of lines of prior evidence, as well as the results of the current study, demonstrate that this is not the case. First, extensive flow cytometry analysis of 123 peripheral immune cell subsets in cancer patients treated in a phase I trial with avelumab (NCT01772004) have shown little, if any, changes in frequency from pre-therapy levels, including in those subsets that express PD-L1, thus demonstrating that this agent can be administered without immune toxicity (manuscript in preparation).^16, 17, 18^ In addition, a recent report has shown that, whereas avelumab efficiently mediates ADCC lysis of human tumor cells that express PD-L1, only nominal levels of lysis were observed when unstimulated PBMCs were used as targets.^15^ In the present study, we have addressed the concern that activated immune cells, which have increased levels of PD-L1,^33^ may become targets of avelumab, and our results suggest this not to be the case. Whereas total PBMC number was increased to a slightly lesser extent when cultures were stimulated with viral peptide pools and treated with avelumab compared with those that received the isotype control, cell viability remained high and unchanged regardless of treatment with avelumab (Table 1). In addition, examination of various immune cell subsets, when PBMCs were stimulated with viral peptides and treated with avelumab, demonstrated a reduction only in the percentage of CD4^+^ T lymphocytes. The decrease in CD4^+^ T cells was consistent with a reduction in proliferation and not an induction of lysis; eight out of nine donors who had a decrease in CD4^+^ lymphocytes also showed a reduction in Ki67 on CD4^+^ T cells (Figure 5a). Furthermore, evaluation of CD4^+^ T-cell number throughout the *in vitro* assay supported the notion that avelumab reduced proliferation of this subset, with CD4^+^ T-cell number remaining low when PBMCs were treated with avelumab compared with the isotype control (Figure 5b). In addition, in our assay, PD-L1 expression was increased following stimulation to a similar extent in CD4^+^ and CD8^+^ T cells, and to a lesser extent than that seen in B lymphocytes (Table 2), suggesting that relative to other subsets CD4^+^ T cells are not more likely to be a target of avelumab-mediated ADCC based on their expression of PD-L1. The reduction in CD4 number at the end of the IVS in CEFT-stimulated HD PBMCs, which were treated with avelumab compared with the isotype control, was also confirmed at the RNA level by nanostring analysis, with significant reductions in the CD4 transcript noted (Figure 6a). Most importantly, we demonstrate in the present study that activated CD8^+^ T lymphocytes, which also displayed increased expression of PD-L1 upon stimulation, were in fact increased, and not decreased, following the addition of avelumab. Taken together, these findings demonstrate that avelumab did not induce lysis of activated human immune cells that express elevated levels of PD-L1, but instead significantly increased antigen-specific immune activation.

Although CD4^+^ T cells are well known to have an essential role in promoting CD8^+^ T-cell responses and the development of memory,^34, 35, 36, 37^ several preclinical studies have reported an association between low CD4^+^ T cells and an increased magnitude of CD8^+^ cytotoxic T lymphocyte responses and antitumor activity.^38, 39^ Two recent reports have also identified a synergistic enhancement of CD8^+^ T-cell function when the PD-1/PD-L1 blockade is combined with depletion of CD4^+^ T cells.^40, 41^ For example, in a mouse model of chronic lymphocytic choriomeningitis virus infection, the combination of PD-L1 blockade with CD4^+^ T-cell depletion rescued exhausted CD8^+^ T cells.^41^ In the current study, we have found that a single agent, anti-PD-L1 avelumab, both reduces the proliferation of CD4^+^ T lymphocytes and enhances CD8^+^ T-cell activation, and that PBMCs from HDs displaying the greatest reduction in CD4^+^ T-cell proliferation also developed the greatest increase in multifunctional CD8^+^ T lymphocytes (Figure 5c). To our knowledge, this study is the first in human cells showing an association between a reduction in CD4^+^ T cells and a corresponding increase in activated CD8^+^ T lymphocytes.

A number of recent preclinical studies in models of infectious disease and cancer have combined the blockade of the PD-1/PD-L1 axis with vaccines to enhance antigen-specific responses.^42, 43, 44^ For example, HIV-infected mice treated with a dendritic cell-directed lentiviral vaccine (DCLV-Gag) in combination with an anti-PD-L1 antibody showed an increase in antigen-specific CD8^+^ T cells that produced multiple cytokines, responded to a broader range of epitopes and had long-lasting memory compared with the monotherapies alone.^42^ Similarly, the combination of a therapeutic adenovirus-based vaccine targeting human papilloma virus with anti-PD-1 blockade in mice bearing human papilloma virus-E6/E7 TC-1 tumors showed an enhanced therapeutic effect.^45^ The finding in the present study that avelumab did not induce lysis of activated human immune cells that express elevated levels of PD-L1, but instead significantly increased antigen-specific immune activation, provides the rationale to incorporate PD-L1 blockade, with a fully functional IgG1 MAb such as avelumab, to enhance the detection of viral/cancer antigen-specific T-cell responses in IVS assays.

## Methods

### HD and cancer patient samples and characteristics

PBMCs were isolated from blood obtained from HDs from the NIH Clinical Center Blood Bank (NCT00001846). Briefly, PBMCs were isolated by Ficoll density gradient separation (Mediatech, Manassas, VA, USA) and cryopreserved at a concentration of 2.5–5 × 10^7^ cells per ml. A total of 17 HDs, nine males and eight females, with a median age of 27 (range, 20–69) and different haplotypes were analyzed in this study. In addition, PBMCs were also obtained from seven patients with metastatic breast cancer with the median age of 55 (range, 40–65) enrolled in a Phase II study at the National Cancer Institute (NCT00179309), with PBMCs used in this study isolated from patients before treatment.

### Peptide pools

Peptide pools of CEFT encoding CMV, EBV, Flu and tetanus toxin, as well as HLA (peptide sequences indicated in Supplementary Table 3) were used as positive and negative stimulators, respectively, and were purchased from JPT (Berlin, Germany). The CEFT pool consists mainly of short peptides (9-mers) and induces mostly CD8 responses. Peptide pools were reconstituted in dimethylsulphoxide (0.625 μg ml^−1^), and added on day 0 and day 11 of the IVS assay at a final concentration of 0.1 μg ml^−1^. Individual 9-mer peptides encoding Flu and HIV-gag, purchased from CPC Scientific (Sunnyvale, CA, USA), were used at a concentration of 0.1 μg ml^−1^.

### Anti-PD-L1-blocking antibodies

Avelumab, a fully humanized IgG1 anti-PD-L1 antibody designed to mediate ADCC of tumor cells, and the corresponding isotype control were obtained from EMD Serono as part of a Cooperative Research and Development Agreement with the Laboratory of Tumor Immunology and Biology, National Cancer Institute, National Institutes of Health. In addition, cell culture grade and endotoxin-free commercially available PD-L1-blocking antibodies clone MIH1 (mouse anti-human IgG1, eBioscience), and clone 29E.2A3 (mouse anti-human, IgG2b,k, Biolegend), as well as corresponding isotype controls were obtained. All PD-L1-blocking antibodies and isotype controls were added at the start of the stimulation assay at a final concentration of 20 μg ml^−1^.

### IVS assay conditions

To measure antigen-specific T-cell responses, we began by optimizing the protocol for the stimulation of PBMCs. PBMCs from HDs (*n*=2) were stimulated for 24 h or 7 days (Supplementary Figure 3a) with 9-mer Flu or HIV-gag (HIV) peptides and were assessed by intracellular cytokine staining for multifunctional CD8^+^ T lymphocytes (producing IFNγ and positive for CD107a). A longer period of stimulation (7 days) enhanced CD8^+^ T lymphocyte activation by 4.4-fold relative to a period of short stimulation (24 h; Supplementary Figure 3b). We also tested the magnitude of response generated by a single 9-mer Flu peptide compared with a pool of peptides (CEFT, mostly 9-mers) encoding CMV, EBV, Flu and tetanus toxin in a 7-day IVS assay, and noted that the frequency of multifunctional CD8^+^ T cells (IFNγ^+^ and CD107a^+^) rose from 0.04% with the single Flu peptide to 1.3% with the CEFT peptide pool (Supplementary Figure 3c). In addition, we examined the effect of a 4-day period of rest, following the 7 days of stimulation and before the restimulation, on the magnitude of response generated by the CEFT peptide pool (Supplementary Figure 3a). Cultures that were rested had a threefold increase in multifunctional CD8^+^ T cells compared with those that were not rested and immediately restimulated following the IVS assay (Supplementary Figure 3d). On the basis of these results, all subsequent assays were performed using the CEFT peptide pool (for positive stimulation) and the HLA peptide pool (as a negative control) in a 7-day IVS assay, followed by a 4-day period of rest before restimulation, as described below.

### IVS assay and intracellular cytokine staining

Cryopreserved PBMCs from HDs were defrosted and rested overnight at 37 °C, 5% CO_2_ in Iscove's DMEM medium (Mediatech) supplemented with 10% heat-inactivated human AB serum (Omega Scientific, Terzana, CA, USA), 2 mM glutamine (Mediatech), 100 units per ml penicillin and 100 μg ml^−1^ streptomycin (Mediatech) at a concentration of 5–10 × 10^6^ cells per ml in a six-well plate. The next day (day 0), PBMCs were harvested, counted with trypan blue exclusion, seeded in 12-well plates (2.5 × 10^6^ cells in 1 ml) and stimulated with 0.1 μg ml^−1^ of a peptide pool of CEFT encoding CMV, EBV, Flu and tetanus toxin, or the negative control peptide pool HLA. Where specified, anti-PD-L1-blocking antibodies or the appropriate isotype controls were added to the cultures on day 0 at a concentration of 20 μg ml^−1^. Cultures were supplemented on days 3 and 5 with cytokines (IL7 and IL15, 10 ng ml^−1^, PeproTech, Rocky Hill, NJ, USA) and fresh medium for a final volume of 2 ml per well, and on day 7 were rested (with the removal of cytokine, peptide and drug). Supernatants were collected on days 5 and 7 and stored at –20 °C. On day 11, PBMCs were harvested by scraping, and 1 × 10^6^ cells were restimulated for 24 h in 96 round-bottom well plates with peptide pools (0.1 μg ml^−1^) in the presence of anti-CD107a-APC (clone H4A3, BD Biosciences, San Jose, CA, USA); brefeldin A (1μl ml^−1^) and monensin (0.7μl ml^−1^; BD Biosciences) were added to cultures 2 h after the start of the restimulation and were incubated for the remaining 22 h.

Following the 24-h restimulation (day 12), PBMCs were incubated for 15 min in Fc block (Biolegend) with Live/Dead Fixable blue (Life Technologies, Frederick, MD, USA) in phosphate-buffered saline with 1% bovine serum albumin. Cells were then stained for 20 min at 4 °C with anti-CD4-PerCP-Cy5.5 (clone OKT4, Biolegend) and anti-CD8-AF700 (clone OKT8, eBioscience), and then fixed and permeabilized according to the manufacturer's instructions (BD Cytofix/Cytoperm kit, BD Biosciences), and stained for 20 min at room temperature with anti-IFNγ-PE-Cy7 (clone 4SB3, BD Biosciences) or anti-IFNγ-BV510 (clone 4S.B3, Biolegend). Antibody concentrations were based on titration experiments. Up to 3 × 10^5^ stained cells were acquired with an LSRII flow cytometer (BD Biosciences) equipped with a UV, violet, blue and red laser. Flow cytometry standard (FCS) files were analyzed using the FlowJo software V9.7 For Macintosh (TreeStar, Ashland, OR, USA). Fluorescence minus one controls were used for gating, and nonviable cells were excluded.

### Evaluation of immune cell subsets

PBMCs were harvested and stained on day 0 (after the overnight rest) and day 12 (following the restimulation) of the IVS assay, and 1 × 10^6^ cells were incubated for 15 min in Fc block (Biolegend) with Live/Dead Fixable blue (Life Technologies) in phosphate-buffered saline with 1% bovine serum albumin. Cells were then stained for 30 min at 4 °C in three panels with the following extracellular antibodies: anti-CD4-FITC (clone OKT4), anti-CD127-APC-Cy7 (clone EBioRDR5) (eBioscience), anti-PD-1-PE (clone MIH4), anti-ki67-PerCP-Cy5.5 (clone B56), anti-PD-L1-PE-Cy7 (clone MIH1), anti-CD25-BV605 (clone 2A3), anti-CD8-AF700 (clone RTA-T8), anti-CD14-V450 (clone MoP9), anti-CD11b-APC-Cy7 (clone ICRF44), anti-CD3-FITC (clone HIT3a), anti-CD11c-APC (clone B-ly6), anti-CD19-APC-H7 (clone SJ25C1) (BD Biosciences), anti-CD15-FITC (clone HI98), anti-HLA-DR-BV605 (clone 243), anti-CD33-APC (clone WM53), anti-CD123-V421 (clone 6H6), anti-CD56-BV605 (clone HCD56) and anti-CD16-APC (clone 3G8; Biolegend). In some cases, CD11c and CD123 were substituted with the following antibodies: anti-CD303-PerCP-Cy5.5 (clone 201a) and anti-CD1c-APC-Cy7 (clone L161; Biolegend). PBMCs were then fixed and permeabilized according to the manufacturer's instructions (FoxP3 Fixation and Permeabilization Kit, eBioscience), and were stained for 30 min at room temperature with anti-FoxP3-eFluor450 (clone 236A/E7, eBioscience). Antibody concentrations were based on titration experiments. Up to 3 × 10^5^ stained cells were acquired with an LSRII flow cytometer (BD Biosciences) equipped with a UV, violet, blue and red laser. FCS files were analyzed using the FlowJo software V9.7 For Macintosh (TreeStar). Fluorescence minus one controls were used for gating, and nonviable cells were excluded.

### Cytokine bead array assay

Supernatants collected on day 7 of the IVS assay, from PBMCs stimulated with CEFT peptides and treated with anti-PD-L1 (avelumab), or the appropriate isotype control, were diluted (1:10) and analyzed for the production of Th1 (IFNγ, TNF and IL2) and Th2 (IL5, IL4 and IL10) cytokines with the Human Cytometric Bead Array Th1/Th2 Cytokine Kit (BD Biosciences). Samples were acquired with a FACS Scan (BD Biosciences) equipped with a red laser, and were analyzed with the Cell Quest Pro software (BD Biosciences).

### Nanostring analyses

PBMCs from six HDs and three breast cancer patients were harvested at the end of the IVS as described above, and were lysed with RLT buffer (Qiagen, Valencia, CA, USA) according to the manufacturer's instructions. Cell lysates containing 100 ng per sample of RNA were hybridized for 17–22 h and run on the nCounter Analysis System (NanoString Technologies, Seattle, WA, USA). Counts were gathered by scanning on HIGH mode for 600 fields of view per sample. Counts were background-subtracted and normalized according to the manufacturer's instructions. Fold changes were calculated using normalized medians of PBMCs that were stimulated with CEFT or HLA and were treated with avelumab or the isotype control. Notable immune-related genes were those identified with trends of differential expression (*P<*0.1 and sorted by fold change) between PBMCs of HDs stimulated with CEFT and treated with avelumab relative to the isotype control.

### Statistical analysis

Statistical analyses were performed with GraphPad Prism 6 (GraphPad Software, La Jolla, CA, USA). Immunological analyses of paired flow cytometry data were performed with the nonparametric Wilcoxon matched-pairs signed rank test. The Mann–Whitney test was used to analyze the difference in immune phenotype before the IVS assay between donors who responded and did not respond to anti-PD-L1 blockade. Correlations between two variables were tested for significance with a Spearman test. *P*-values <0.05 were considered significant. For nanostring analyses, *P-*values were calculated using the Wilcoxon matched-pairs signed rank test.

## Figures and Tables

**Table 1:** Effect of anti-PD-L1 (avelumab) on cell number, viability and frequency of immune cell subsets

*Subset measured*							P*-value of anti-PD-L1 versus isotype control in HLA*	P*-value of anti-PD-L1 versus isotype control in CEFT*
	*HLA*			*CEFT*				
	*No treatment*	*Isotype control*	*Anti-PD-L1*	*No treatment*	*Isotype control*	*Anti-PD-L1*		
*n*=17								
Total cell count	4.5 (3.21–6.35)	4.82 (3.78–6.65)	3.66 (2.49–5.13)	5.23 (4.23–6.58)	5.05 (4.37–6.65)	4.6 (3.61–5.85)	**0.0009**	**0.0052**
Viability	69 (57–88)	73 (62–89)	67 (57–94)	66 (54–93)	62 (59–93)	68 (58–96)	0.3529	0.2435
CD8	27.9 (18.7–45.4)	28.6 (18.1–43.2)	30.2 (25.1–47.2)	36.9 (23.5–43.6)	26.6 (23.2–43.2)	35.3 (28.3–46.1)	**0.02**	0.15
CD4	49.5 (37.7–61.8)	47.3 (40.7–59.6)	42.5 (30.1–49.4)	50.9 (34.4–59.8)	48.3 (33.0–59.2)	26.7 (18.1–40.7)	**0.001**	**<0.0001**
*n*=5								
B cells	4.5 (2.05–8.3)	4.3 (2.3–9.2)	6.7 (1.8–10.25)	2.10 (1.35–7.50)	1.60 (1.20–6.55)	6.5 (1.6–8.7)	0.88	0.06
NK-T cells	3.6 (1.15–4.35)	4.5 (1.15–5.45)	2.1 (1.4–4.35)	0.9 (0.45–2.45)	1.30 (0.60–2.90)	1.10 (0.50–2.95)	0.81	>0.99
NK cells	8.3 (3.65–13.2)	2.8 (1.4–4.1)	1.8 (0.55–3.75)	1.60 (0.75–3.45)	2.00 (1.30–2.90)	1.60 (1.10–4.90)	0.19	>0.99
Treg	0.52 (0.25–0.69)	0.51 (0.34–0.77)	0.26 (0.16–0.38)	0.53 (0.30–0.73)	0.34 (0.23–0.61)	0.15 (0.03–0.40)	0.19	0.19

Abbreviations: HLA, human leukocyte antigen; IVS, *in vitro* stimulation; NK, natural killer; PBMC, peripheral blood mononuclear cell; PD-L1, programmed death-ligand 1; Treg, T regulatory cells.

PBMCs from healthy donors (*n*=5–17) that were stimulated with CEFT or HLA peptide pools, and treated with avelumab or the isotype control, as described in Supplementary Figure 3a, were harvested on day 12 of the IVS assay and were analyzed with trypan blue exclusion and flow cytometry to evaluate the effects of avelumab on cell number, viability and frequency of immune cell subsets. Values represent the medians of cell number, or the percentage of PBMCs that were viable, as well as the median frequency of CD8^+^ and CD4^+^ T lymphocytes, B cells (CD19^+^), NK-T cells (CD3^+^, CD56^+^), NK cells (CD3^−^, CD56^+^), and Tregs (CD4^+^, CD25^+^, FoxP3^+^, CD127^−^). Interquartile ranges are indicated in parentheses. Data were analyzed with the Wilcoxon matched-pairs signed rank test, with *P*-values indicated. *P*-values< 0.05 are indicated in bold.

**Table 2:** PD-1 and PD-L1 expression in immune cell subsets at the end of *in vitro* stimulation with peptides in the absence of anti-PD-L1 treatment

*Subsets measured*	*HLA*	*CEFT*
CD8 PD-L1^+^	0.82 (0.6–1.42)	1.65 (1.07–2.32)
CD8 PD-1^+^	0.46 (0.12–0.98)	1.18 (0.4–2.17)
CD4 PD-L1^+^	0.72 (0.55–0.89)	1.17 (1.11–1.46)
CD4 PD-1^+^	1.68 (0.8–2.31)	2.52 (2.11–2.89)
B cell PD-L1^+^	3.1 (2.75–11.3)	11.3 (5.63–17.8)
B cell PD-1^+^	1.5 (0.9–5.48)	7.45 (3.75–9.58)
CD8 IFNγ^+^ PD-L1^+^	0.01 (0.0–0.02)	0.25 (0.1–0.57)
CD8 IFNγ^+^ PD-1^+^	0.0 (0.0–0.03)	1.15 (0.08–1.43)
CD8 CD107a^+^ PD-L1^+^	0.03 (0.01–0.06)	0.23 (0.1–0.36)
CD8 CD107a^+^ PD-1^+^	0.01 (0.0–0.1)	0.22 (0.1–1.19)
CD8 CD107a^+^ IFNγ^+^ PD-L1^+^	0.0 (0.0–0.02)	0.14 (0.05–0.21)
CD8 CD107a^+^ IFNγ^+^ PD-1^+^	0.0 (0.0–0.02)	0.05 (0.03–0.71)

Abbreviations: HLA, human leukocyte antigen; PBMC, peripheral blood mononuclear cell; PD-1, programmed death-1; PD-L1, programmed death-ligand 1.

PBMCs from healthy donors (n=4), stimulated with CEFT or HLA, as described in Supplementary Figure 3a (without avelumab), were harvested on day 12 of the stimulation assay and analyzed by flow cytometry for PD-1 (clone MIH4) and PD-L1 (clone MIH1) expression. Values represent the median percentage of PBMCs that were CD8^+^, CD4^+^ or CD19^+^ (B cells) and expressed PD-1 or PD-L1, or activated CD8^+^ T cells (IFNγ^+^, CD107a^+^ and CD107a^+^ IFNγ^+^) that expressed PD-1 or PD-L1 as a percentage of total CD8^+^ T lymphocytes. Interquartile ranges are indicated in parentheses.
