# Supplementary material for: A fully human IgG1 anti-PD-L1 MAb in an in vitro assay enhances antigen-specific T-cell responses
Source: Clin Transl Immunology. 2016 May 20;5(5):e83–. doi: 10.1038/cti.2016.27 (PMC4910121; doi:10.1038/cti.2016.27)
Supplement: Supplementary Figure 1 [file cti201627x1.ppt]

## Slide 1
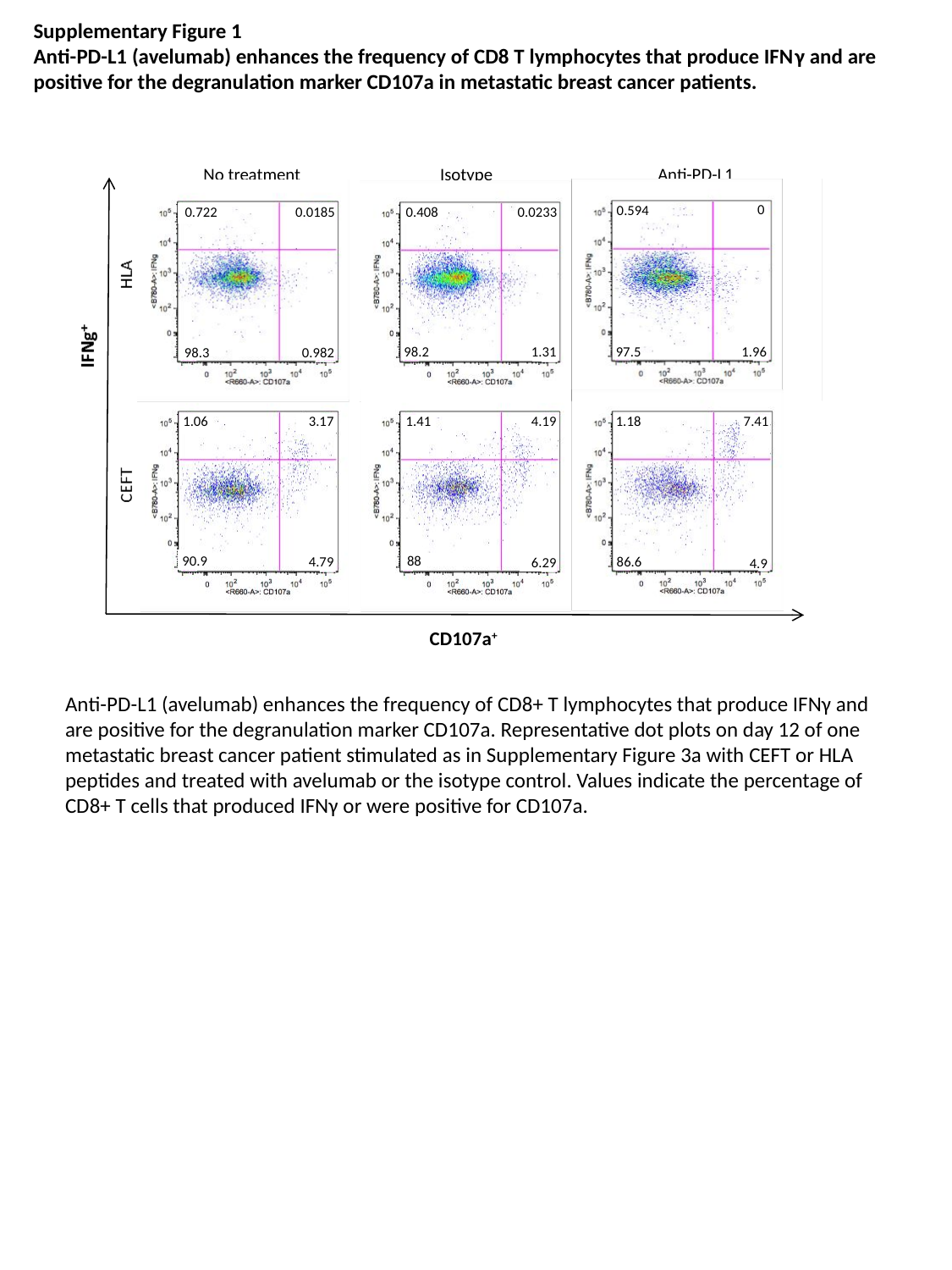

# Supplementary Figure 1 Anti-PD-L1 (avelumab) enhances the frequency of CD8 T lymphocytes that produce IFNγ and are positive for the degranulation marker CD107a in metastatic breast cancer patients.
No treatment
Isotype
Anti-PD-L1
0
0.594
0.722
0.0185
0.408
0.0233
97.5
1.96
98.2
1.31
98.3
0.982
1.06
3.17
4.19
1.18
7.41
1.41
90.9
88
4.79
86.6
6.29
4.9
CD107a+
Anti-PD-L1 (avelumab) enhances the frequency of CD8+ T lymphocytes that produce IFNγ and are positive for the degranulation marker CD107a. Representative dot plots on day 12 of one metastatic breast cancer patient stimulated as in Supplementary Figure 3a with CEFT or HLA peptides and treated with avelumab or the isotype control. Values indicate the percentage of CD8+ T cells that produced IFNγ or were positive for CD107a.
