# Supplementary material for: A fully human IgG1 anti-PD-L1 MAb in an in vitro assay enhances antigen-specific T-cell responses
Source: Clin Transl Immunology. 2016 May 20;5(5):e83–. doi: 10.1038/cti.2016.27 (PMC4910121; doi:10.1038/cti.2016.27)
Supplement: Supplementary Figure 2 [file cti201627x2.ppt]

## Slide 1
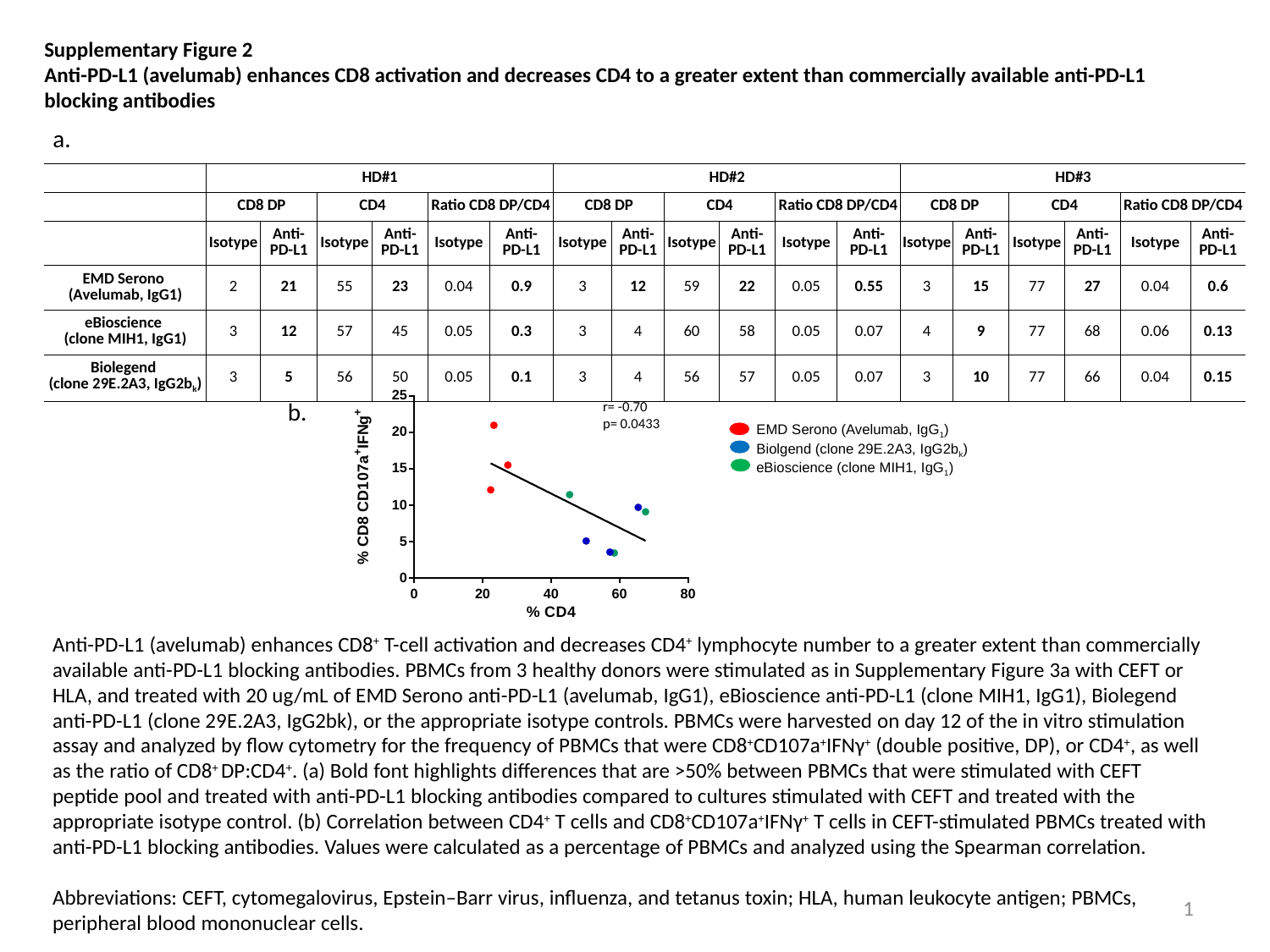

# Supplementary Figure 2 Anti-PD-L1 (avelumab) enhances CD8 activation and decreases CD4 to a greater extent than commercially available anti-PD-L1 blocking antibodies
a.
| | HD#1 | | | | | | HD#2 | | | | | | HD#3 | | | | | |
| --- | --- | --- | --- | --- | --- | --- | --- | --- | --- | --- | --- | --- | --- | --- | --- | --- | --- | --- |
| | CD8 DP | | CD4 | | Ratio CD8 DP/CD4 | | CD8 DP | | CD4 | | Ratio CD8 DP/CD4 | | CD8 DP | | CD4 | | Ratio CD8 DP/CD4 | |
| | Isotype | Anti-PD-L1 | Isotype | Anti-PD-L1 | Isotype | Anti- PD-L1 | Isotype | Anti-PD-L1 | Isotype | Anti-PD-L1 | Isotype | Anti- PD-L1 | Isotype | Anti-PD-L1 | Isotype | Anti-PD-L1 | Isotype | Anti- PD-L1 |
| EMD Serono (Avelumab, IgG1) | 2 | 21 | 55 | 23 | 0.04 | 0.9 | 3 | 12 | 59 | 22 | 0.05 | 0.55 | 3 | 15 | 77 | 27 | 0.04 | 0.6 |
| eBioscience (clone MIH1, IgG1) | 3 | 12 | 57 | 45 | 0.05 | 0.3 | 3 | 4 | 60 | 58 | 0.05 | 0.07 | 4 | 9 | 77 | 68 | 0.06 | 0.13 |
| Biolegend (clone 29E.2A3, IgG2bk) | 3 | 5 | 56 | 50 | 0.05 | 0.1 | 3 | 4 | 56 | 57 | 0.05 | 0.07 | 3 | 10 | 77 | 66 | 0.04 | 0.15 |
b.
r= -0.70
p= 0.0433
EMD Serono (Avelumab, IgG1)
Biolgend (clone 29E.2A3, IgG2bk)
eBioscience (clone MIH1, IgG1)
Anti-PD-L1 (avelumab) enhances CD8+ T-cell activation and decreases CD4+ lymphocyte number to a greater extent than commercially available anti-PD-L1 blocking antibodies. PBMCs from 3 healthy donors were stimulated as in Supplementary Figure 3a with CEFT or HLA, and treated with 20 ug/mL of EMD Serono anti-PD-L1 (avelumab, IgG1), eBioscience anti-PD-L1 (clone MIH1, IgG1), Biolegend anti-PD-L1 (clone 29E.2A3, IgG2bk), or the appropriate isotype controls. PBMCs were harvested on day 12 of the in vitro stimulation assay and analyzed by flow cytometry for the frequency of PBMCs that were CD8+CD107a+IFNγ+ (double positive, DP), or CD4+, as well as the ratio of CD8+ DP:CD4+. (a) Bold font highlights differences that are >50% between PBMCs that were stimulated with CEFT peptide pool and treated with anti-PD-L1 blocking antibodies compared to cultures stimulated with CEFT and treated with the appropriate isotype control. (b) Correlation between CD4+ T cells and CD8+CD107a+IFNγ+ T cells in CEFT-stimulated PBMCs treated with anti-PD-L1 blocking antibodies. Values were calculated as a percentage of PBMCs and analyzed using the Spearman correlation.
Abbreviations: CEFT, cytomegalovirus, Epstein–Barr virus, influenza, and tetanus toxin; HLA, human leukocyte antigen; PBMCs, peripheral blood mononuclear cells.
<number>
