# Supplementary material for: A fully human IgG1 anti-PD-L1 MAb in an in vitro assay enhances antigen-specific T-cell responses
Source: Clin Transl Immunology. 2016 May 20;5(5):e83–. doi: 10.1038/cti.2016.27 (PMC4910121; doi:10.1038/cti.2016.27)
Supplement: Supplementary Figure 3 [file cti201627x3.ppt]

## Slide 1
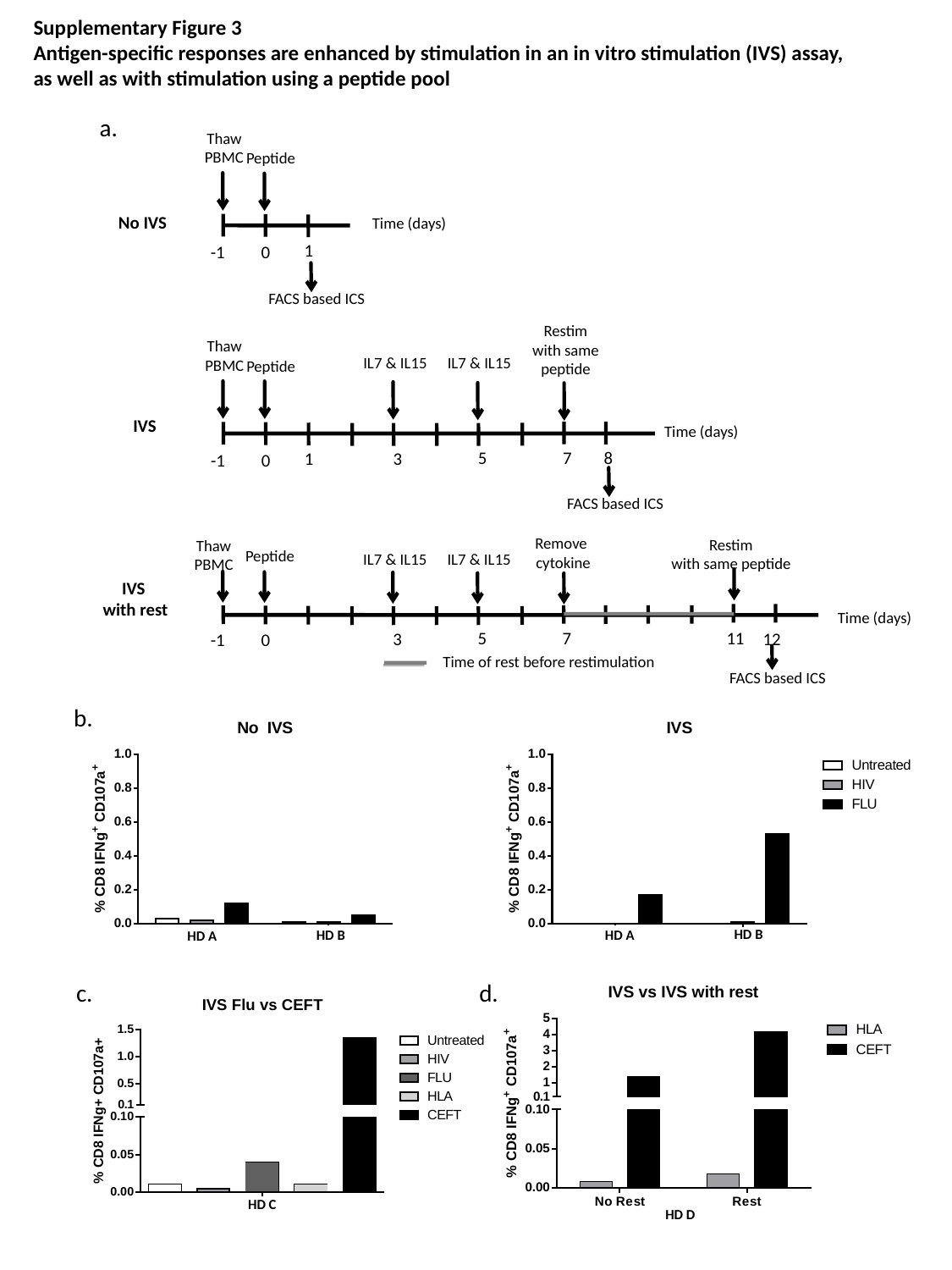

# Supplementary Figure 3 Antigen-specific responses are enhanced by stimulation in an in vitro stimulation (IVS) assay, as well as with stimulation using a peptide pool
a.
Thaw PBMC
Peptide
Time (days)
1
-1
0
FACS based ICS
No IVS
Restim
with same
peptide
Thaw PBMC
IL7 & IL15
IL7 & IL15
Peptide
Time (days)
7
8
5
1
3
-1
0
FACS based ICS
IVS
Remove
cytokine
Restim
with same peptide
Peptide
IL7 & IL15
IL7 & IL15
Time (days)
7
11
5
12
3
-1
0
Time of rest before restimulation
FACS based ICS
Thaw PBMC
IVS
with rest
b.
HD B
HD B
HD A
HD A
c.
d.
HD C
HD D

## Slide 2
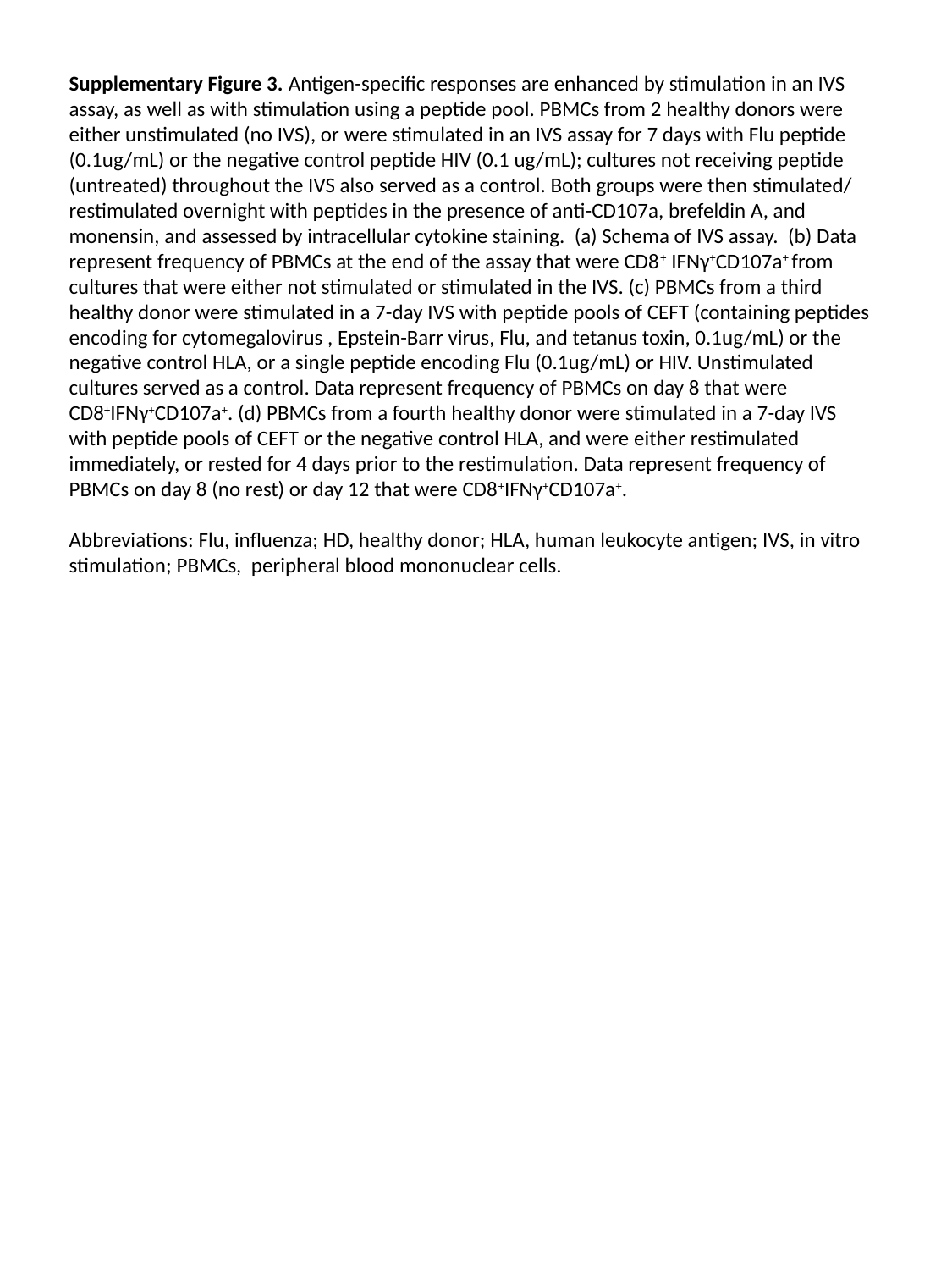

Supplementary Figure 3. Antigen-specific responses are enhanced by stimulation in an IVS assay, as well as with stimulation using a peptide pool. PBMCs from 2 healthy donors were either unstimulated (no IVS), or were stimulated in an IVS assay for 7 days with Flu peptide (0.1ug/mL) or the negative control peptide HIV (0.1 ug/mL); cultures not receiving peptide (untreated) throughout the IVS also served as a control. Both groups were then stimulated/ restimulated overnight with peptides in the presence of anti-CD107a, brefeldin A, and monensin, and assessed by intracellular cytokine staining. (a) Schema of IVS assay. (b) Data represent frequency of PBMCs at the end of the assay that were CD8+ IFNγ+CD107a+ from cultures that were either not stimulated or stimulated in the IVS. (c) PBMCs from a third healthy donor were stimulated in a 7-day IVS with peptide pools of CEFT (containing peptides encoding for cytomegalovirus , Epstein-Barr virus, Flu, and tetanus toxin, 0.1ug/mL) or the negative control HLA, or a single peptide encoding Flu (0.1ug/mL) or HIV. Unstimulated cultures served as a control. Data represent frequency of PBMCs on day 8 that were CD8+IFNγ+CD107a+. (d) PBMCs from a fourth healthy donor were stimulated in a 7-day IVS with peptide pools of CEFT or the negative control HLA, and were either restimulated immediately, or rested for 4 days prior to the restimulation. Data represent frequency of PBMCs on day 8 (no rest) or day 12 that were CD8+IFNγ+CD107a+.
Abbreviations: Flu, influenza; HD, healthy donor; HLA, human leukocyte antigen; IVS, in vitro stimulation; PBMCs, peripheral blood mononuclear cells.
