# Supplementary material for: A fully human IgG1 anti-PD-L1 MAb in an in vitro assay enhances antigen-specific T-cell responses
Source: Clin Transl Immunology. 2016 May 20;5(5):e83–. doi: 10.1038/cti.2016.27 (PMC4910121; doi:10.1038/cti.2016.27)
Supplement: Supplementary Table 1 [file cti201627x4.ppt]

## Slide 1
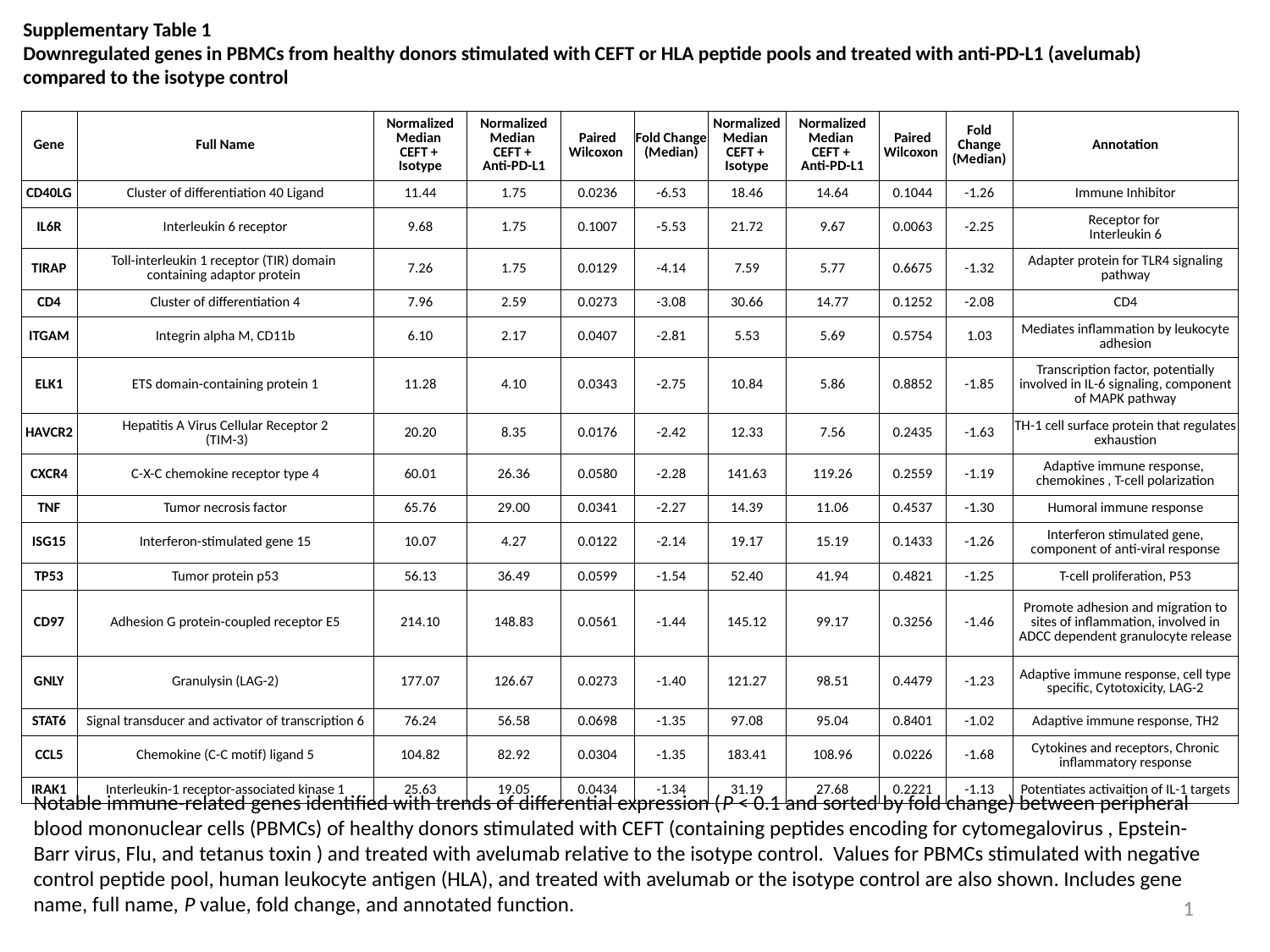

Supplementary Table 1
Downregulated genes in PBMCs from healthy donors stimulated with CEFT or HLA peptide pools and treated with anti-PD-L1 (avelumab) compared to the isotype control
| Gene | Full Name | Normalized Median CEFT + Isotype | Normalized Median CEFT + Anti-PD-L1 | Paired Wilcoxon | Fold Change (Median) | Normalized Median CEFT + Isotype | Normalized Median CEFT + Anti-PD-L1 | Paired Wilcoxon | Fold Change (Median) | Annotation |
| --- | --- | --- | --- | --- | --- | --- | --- | --- | --- | --- |
| CD40LG | Cluster of differentiation 40 Ligand | 11.44 | 1.75 | 0.0236 | -6.53 | 18.46 | 14.64 | 0.1044 | -1.26 | Immune Inhibitor |
| IL6R | Interleukin 6 receptor | 9.68 | 1.75 | 0.1007 | -5.53 | 21.72 | 9.67 | 0.0063 | -2.25 | Receptor for Interleukin 6 |
| TIRAP | Toll-interleukin 1 receptor (TIR) domain containing adaptor protein | 7.26 | 1.75 | 0.0129 | -4.14 | 7.59 | 5.77 | 0.6675 | -1.32 | Adapter protein for TLR4 signaling pathway |
| CD4 | Cluster of differentiation 4 | 7.96 | 2.59 | 0.0273 | -3.08 | 30.66 | 14.77 | 0.1252 | -2.08 | CD4 |
| ITGAM | Integrin alpha M, CD11b | 6.10 | 2.17 | 0.0407 | -2.81 | 5.53 | 5.69 | 0.5754 | 1.03 | Mediates inflammation by leukocyte adhesion |
| ELK1 | ETS domain-containing protein 1 | 11.28 | 4.10 | 0.0343 | -2.75 | 10.84 | 5.86 | 0.8852 | -1.85 | Transcription factor, potentially involved in IL-6 signaling, component of MAPK pathway |
| HAVCR2 | Hepatitis A Virus Cellular Receptor 2 (TIM-3) | 20.20 | 8.35 | 0.0176 | -2.42 | 12.33 | 7.56 | 0.2435 | -1.63 | TH-1 cell surface protein that regulates exhaustion |
| CXCR4 | C-X-C chemokine receptor type 4 | 60.01 | 26.36 | 0.0580 | -2.28 | 141.63 | 119.26 | 0.2559 | -1.19 | Adaptive immune response, chemokines , T-cell polarization |
| TNF | Tumor necrosis factor | 65.76 | 29.00 | 0.0341 | -2.27 | 14.39 | 11.06 | 0.4537 | -1.30 | Humoral immune response |
| ISG15 | Interferon-stimulated gene 15 | 10.07 | 4.27 | 0.0122 | -2.14 | 19.17 | 15.19 | 0.1433 | -1.26 | Interferon stimulated gene, component of anti-viral response |
| TP53 | Tumor protein p53 | 56.13 | 36.49 | 0.0599 | -1.54 | 52.40 | 41.94 | 0.4821 | -1.25 | T-cell proliferation, P53 |
| CD97 | Adhesion G protein-coupled receptor E5 | 214.10 | 148.83 | 0.0561 | -1.44 | 145.12 | 99.17 | 0.3256 | -1.46 | Promote adhesion and migration to sites of inflammation, involved in ADCC dependent granulocyte release |
| GNLY | Granulysin (LAG-2) | 177.07 | 126.67 | 0.0273 | -1.40 | 121.27 | 98.51 | 0.4479 | -1.23 | Adaptive immune response, cell type specific, Cytotoxicity, LAG-2 |
| STAT6 | Signal transducer and activator of transcription 6 | 76.24 | 56.58 | 0.0698 | -1.35 | 97.08 | 95.04 | 0.8401 | -1.02 | Adaptive immune response, TH2 |
| CCL5 | Chemokine (C-C motif) ligand 5 | 104.82 | 82.92 | 0.0304 | -1.35 | 183.41 | 108.96 | 0.0226 | -1.68 | Cytokines and receptors, Chronic inflammatory response |
| IRAK1 | Interleukin-1 receptor-associated kinase 1 | 25.63 | 19.05 | 0.0434 | -1.34 | 31.19 | 27.68 | 0.2221 | -1.13 | Potentiates activaition of IL-1 targets |
Notable immune-related genes identified with trends of differential expression (P < 0.1 and sorted by fold change) between peripheral blood mononuclear cells (PBMCs) of healthy donors stimulated with CEFT (containing peptides encoding for cytomegalovirus , Epstein-Barr virus, Flu, and tetanus toxin ) and treated with avelumab relative to the isotype control. Values for PBMCs stimulated with negative control peptide pool, human leukocyte antigen (HLA), and treated with avelumab or the isotype control are also shown. Includes gene name, full name, P value, fold change, and annotated function.
<number>
