# Supplementary material for: A fully human IgG1 anti-PD-L1 MAb in an in vitro assay enhances antigen-specific T-cell responses
Source: Clin Transl Immunology. 2016 May 20;5(5):e83–. doi: 10.1038/cti.2016.27 (PMC4910121; doi:10.1038/cti.2016.27)
Supplement: Supplementary Table 2 [file cti201627x5.ppt]

## Slide 1
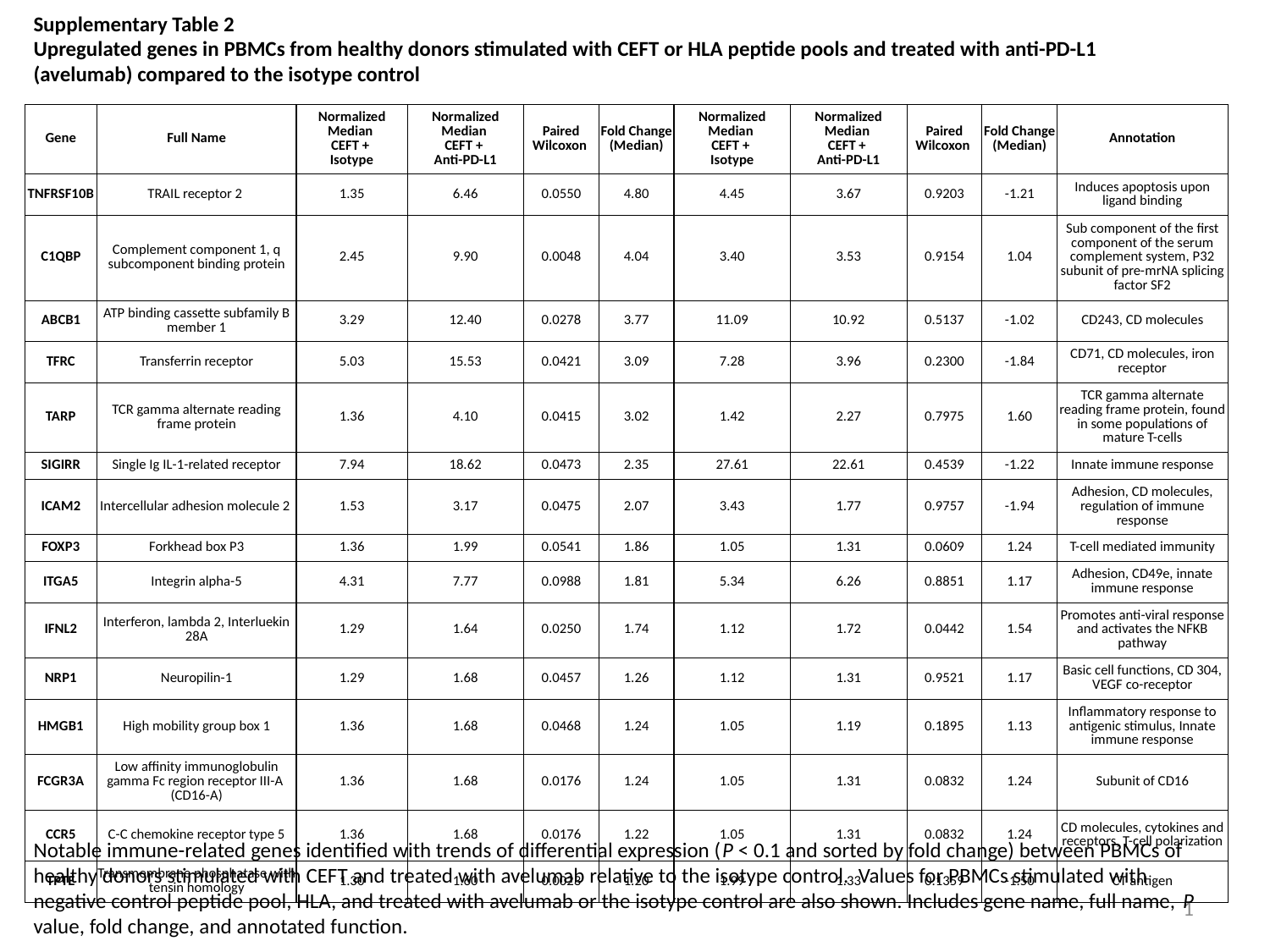

# Supplementary Table 2 Upregulated genes in PBMCs from healthy donors stimulated with CEFT or HLA peptide pools and treated with anti-PD-L1 (avelumab) compared to the isotype control
| Gene | Full Name | Normalized Median CEFT + Isotype | Normalized Median CEFT + Anti-PD-L1 | Paired Wilcoxon | Fold Change (Median) | Normalized Median CEFT + Isotype | Normalized Median CEFT + Anti-PD-L1 | Paired Wilcoxon | Fold Change (Median) | Annotation |
| --- | --- | --- | --- | --- | --- | --- | --- | --- | --- | --- |
| TNFRSF10B | TRAIL receptor 2 | 1.35 | 6.46 | 0.0550 | 4.80 | 4.45 | 3.67 | 0.9203 | -1.21 | Induces apoptosis upon ligand binding |
| C1QBP | Complement component 1, q subcomponent binding protein | 2.45 | 9.90 | 0.0048 | 4.04 | 3.40 | 3.53 | 0.9154 | 1.04 | Sub component of the first component of the serum complement system, P32 subunit of pre-mrNA splicing factor SF2 |
| ABCB1 | ATP binding cassette subfamily B member 1 | 3.29 | 12.40 | 0.0278 | 3.77 | 11.09 | 10.92 | 0.5137 | -1.02 | CD243, CD molecules |
| TFRC | Transferrin receptor | 5.03 | 15.53 | 0.0421 | 3.09 | 7.28 | 3.96 | 0.2300 | -1.84 | CD71, CD molecules, iron receptor |
| TARP | TCR gamma alternate reading frame protein | 1.36 | 4.10 | 0.0415 | 3.02 | 1.42 | 2.27 | 0.7975 | 1.60 | TCR gamma alternate reading frame protein, found in some populations of mature T-cells |
| SIGIRR | Single Ig IL-1-related receptor | 7.94 | 18.62 | 0.0473 | 2.35 | 27.61 | 22.61 | 0.4539 | -1.22 | Innate immune response |
| ICAM2 | Intercellular adhesion molecule 2 | 1.53 | 3.17 | 0.0475 | 2.07 | 3.43 | 1.77 | 0.9757 | -1.94 | Adhesion, CD molecules, regulation of immune response |
| FOXP3 | Forkhead box P3 | 1.36 | 1.99 | 0.0541 | 1.86 | 1.05 | 1.31 | 0.0609 | 1.24 | T-cell mediated immunity |
| ITGA5 | Integrin alpha-5 | 4.31 | 7.77 | 0.0988 | 1.81 | 5.34 | 6.26 | 0.8851 | 1.17 | Adhesion, CD49e, innate immune response |
| IFNL2 | Interferon, lambda 2, Interluekin 28A | 1.29 | 1.64 | 0.0250 | 1.74 | 1.12 | 1.72 | 0.0442 | 1.54 | Promotes anti-viral response and activates the NFKB pathway |
| NRP1 | Neuropilin-1 | 1.29 | 1.68 | 0.0457 | 1.26 | 1.12 | 1.31 | 0.9521 | 1.17 | Basic cell functions, CD 304, VEGF co-receptor |
| HMGB1 | High mobility group box 1 | 1.36 | 1.68 | 0.0468 | 1.24 | 1.05 | 1.19 | 0.1895 | 1.13 | Inflammatory response to antigenic stimulus, Innate immune response |
| FCGR3A | Low affinity immunoglobulin gamma Fc region receptor III-A  (CD16-A) | 1.36 | 1.68 | 0.0176 | 1.24 | 1.05 | 1.31 | 0.0832 | 1.24 | Subunit of CD16 |
| CCR5 | C-C chemokine receptor type 5 | 1.36 | 1.68 | 0.0176 | 1.22 | 1.05 | 1.31 | 0.0832 | 1.24 | CD molecules, cytokines and receptors, T-cell polarization |
| TPTE | Transmembrane phosphatase with tensin homology | 1.30 | 1.60 | 0.0023 | 1.20 | 1.99 | 1.33 | 0.1359 | -1.50 | CT antigen |
Notable immune-related genes identified with trends of differential expression (P < 0.1 and sorted by fold change) between PBMCs of healthy donors stimulated with CEFT and treated with avelumab relative to the isotype control. Values for PBMCs stimulated with negative control peptide pool, HLA, and treated with avelumab or the isotype control are also shown. Includes gene name, full name, P value, fold change, and annotated function.
<number>
