# Supplementary material for: A fully human IgG1 anti-PD-L1 MAb in an in vitro assay enhances antigen-specific T-cell responses
Source: Clin Transl Immunology. 2016 May 20;5(5):e83–. doi: 10.1038/cti.2016.27 (PMC4910121; doi:10.1038/cti.2016.27)
Supplement: Supplementary Table 3 [file cti201627x6.ppt]

## Slide 1
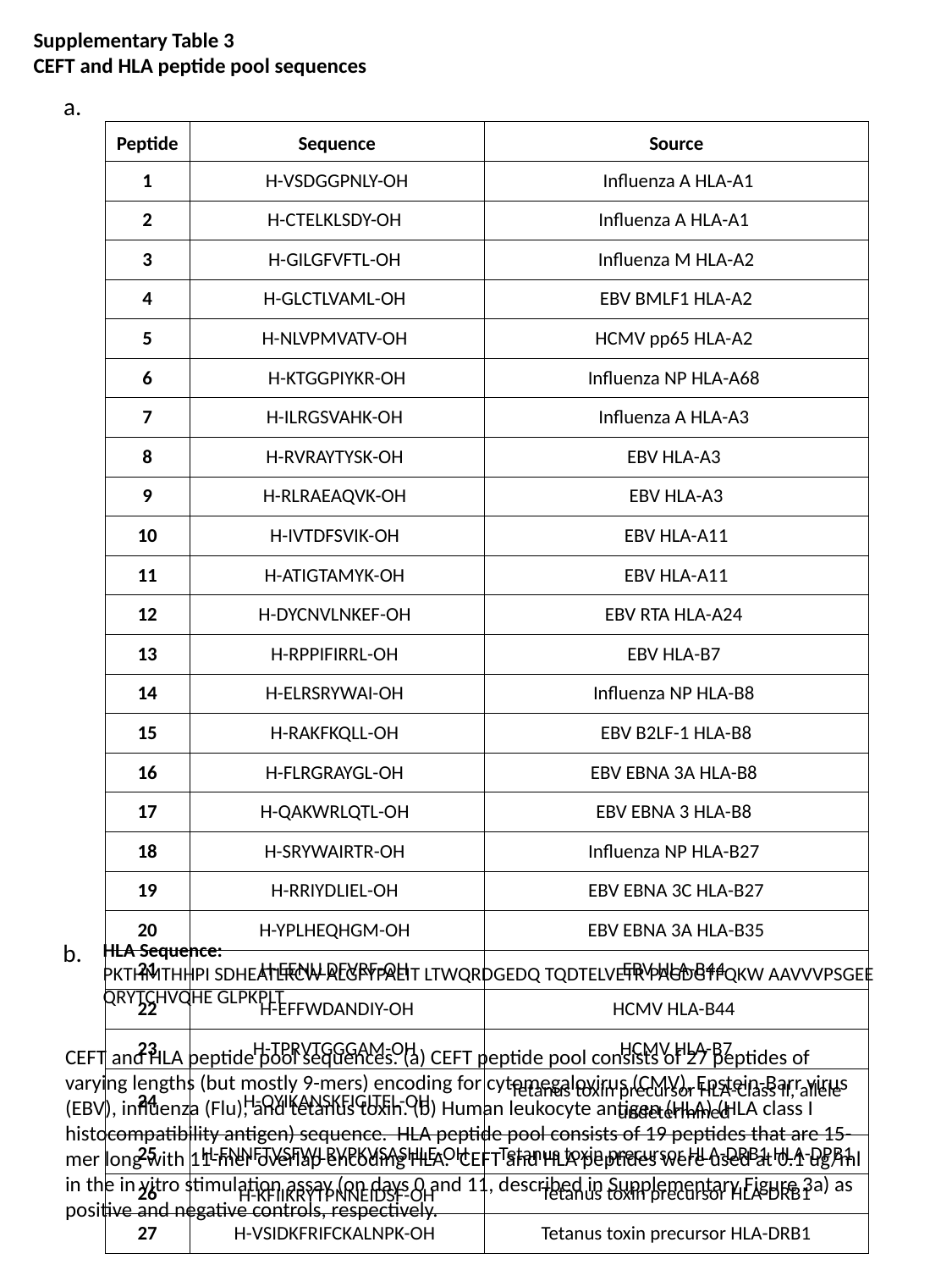

# Supplementary Table 3 CEFT and HLA peptide pool sequences
a.
| Peptide | Sequence | Source |
| --- | --- | --- |
| 1 | H-VSDGGPNLY-OH | Influenza A HLA-A1 |
| 2 | H-CTELKLSDY-OH | Influenza A HLA-A1 |
| 3 | H-GILGFVFTL-OH | Influenza M HLA-A2 |
| 4 | H-GLCTLVAML-OH | EBV BMLF1 HLA-A2 |
| 5 | H-NLVPMVATV-OH | HCMV pp65 HLA-A2 |
| 6 | H-KTGGPIYKR-OH | Influenza NP HLA-A68 |
| 7 | H-ILRGSVAHK-OH | Influenza A HLA-A3 |
| 8 | H-RVRAYTYSK-OH | EBV HLA-A3 |
| 9 | H-RLRAEAQVK-OH | EBV HLA-A3 |
| 10 | H-IVTDFSVIK-OH | EBV HLA-A11 |
| 11 | H-ATIGTAMYK-OH | EBV HLA-A11 |
| 12 | H-DYCNVLNKEF-OH | EBV RTA HLA-A24 |
| 13 | H-RPPIFIRRL-OH | EBV HLA-B7 |
| 14 | H-ELRSRYWAI-OH | Influenza NP HLA-B8 |
| 15 | H-RAKFKQLL-OH | EBV B2LF-1 HLA-B8 |
| 16 | H-FLRGRAYGL-OH | EBV EBNA 3A HLA-B8 |
| 17 | H-QAKWRLQTL-OH | EBV EBNA 3 HLA-B8 |
| 18 | H-SRYWAIRTR-OH | Influenza NP HLA-B27 |
| 19 | H-RRIYDLIEL-OH | EBV EBNA 3C HLA-B27 |
| 20 | H-YPLHEQHGM-OH | EBV EBNA 3A HLA-B35 |
| 21 | H-EENLLDFVRF-OH | EBV HLA-B44 |
| 22 | H-EFFWDANDIY-OH | HCMV HLA-B44 |
| 23 | H-TPRVTGGGAM-OH | HCMV HLA-B7 |
| 24 | H-QYIKANSKFIGITEL-OH | Tetanus toxin precursor HLA-Class II, allele undetermined |
| 25 | H-FNNFTVSFWLRVPKVSASHLE-OH | Tetanus toxin precursor HLA-DRB1 HLA-DPB1 |
| 26 | H-KFIIKRYTPNNEIDSF-OH | Tetanus toxin precursor HLA-DRB1 |
| 27 | H-VSIDKFRIFCKALNPK-OH | Tetanus toxin precursor HLA-DRB1 |
b.
HLA Sequence:
PKTHMTHHPI SDHEATLRCW ALGFYPAEIT LTWQRDGEDQ TQDTELVETR PAGDGTFQKW AAVVVPSGEE QRYTCHVQHE GLPKPLT
CEFT and HLA peptide pool sequences. (a) CEFT peptide pool consists of 27 peptides of varying lengths (but mostly 9-mers) encoding for cytomegalovirus (CMV), Epstein-Barr virus (EBV), influenza (Flu), and tetanus toxin. (b) Human leukocyte antigen (HLA) (HLA class I histocompatibility antigen) sequence. HLA peptide pool consists of 19 peptides that are 15-mer long with 11-mer overlap encoding HLA. CEFT and HLA peptides were used at 0.1 ug/ml in the in vitro stimulation assay (on days 0 and 11, described in Supplementary Figure 3a) as positive and negative controls, respectively.
